# Current Advances in Thyroid Cancer Management. Are We Ready for the Epidemic Rise of Diagnoses?

**DOI:** 10.3390/ijms18081817

**Published:** 2017-08-22

**Authors:** Dagmara Rusinek, Ewa Chmielik, Jolanta Krajewska, Michal Jarzab, Malgorzata Oczko-Wojciechowska, Agnieszka Czarniecka, Barbara Jarzab

**Affiliations:** 1 Department of Nuclear Medicine and Endocrine Oncology, Maria Sklodowska-Curie Memorial Institute-Cancer Center, Gliwice Branch, Wybrzeze Armii Krajowej 15, 44-101 Gliwice, Poland; Dagmara.Rusinek@io.gliwice.pl (D.R.); Jolanta.Krajewska@io.gliwice.pl (J.K.); Malgorzata.Oczko-Wojciechowska@io.gliwice.pl (M.O.-W.); 2 Tumor Pathology Department, Maria Sklodowska-Curie Memorial Institute-Cancer Center, Gliwice Branch, Wybrzeze Armii Krajowej 15, 44-101 Gliwice, Poland; ewachmielik@gmail.com; 3 3rd Department of Radiotherapy and Chemotherapy, Breast Unit, Maria Sklodowska-Curie Memorial Institute-Cancer Center, Gliwice Branch, Wybrzeze Armii Krajowej 15, 44-101 Gliwice, Poland; Michal.Jarzab@io.gliwice.pl; 4 Department of Oncological and Reconstructive Surgery, Maria Sklodowska-Curie Memorial Institute-Cancer Center, Gliwice Branch, Wybrzeze Armii Krajowej 15, 44-101 Gliwice, Poland; Agnieszka.Czarniecka@io.gliwice.pl

**Keywords:** differentiated thyroid cancer, medullary thyroid cancer, anaplastic thyroid cancer, molecular diagnostics, thyroid surgery, RAI (radioiodine) ablation, multi kinase inhibitors

## Abstract

A rising incidence of thyroid cancers (TCs) mainly small tumors, observed during recent years, lead to many controversies regarding treatment strategies. TCs represent a distinct molecular background and clinical outcome. Although in most cases TCs are characterized by a good prognosis, there are some aggressive forms, which do not respond to standard treatment. There are still some questions, which have to be resolved to avoid dangerous simplifications in the clinical management. In this article, we focused on the current advantages in preoperative molecular diagnostic tests and histopathological examination including noninvasive follicular thyroid neoplasm with papillary-like nuclear features (NIFTP). We discussed the controversies regarding the extent of thyroid surgery and adjuvant radioiodine therapy, as well as new treatment modalities for radioiodine-refractory differentiated thyroid cancer (RR-DTC). Considering medullary thyroid cancer (MTC), we analyzed a clinical management based on histopathology and *RET* (ret proto-oncogene) mutation genotype, disease follow-up with a special attention to serum calcitonin doubling time as an important prognostic marker, and targeted therapy applied in advanced MTC. In addition, we provided some data regarding anaplastic thyroid cancer (ATC), a highly lethal neoplasm, which lead to death in nearly 100% of patients due to the lack of effective treatment options.

## 1. Introduction

Thyroid cancer (TC) incidence has demonstrated a rapid growth during the last few decades. According to the National Cancer Institute Surveillance, Epidemiology, and End Results (SEER) Program, TC represents 3.4% of all cancers diagnosed yearly [1]. Rahib et al. estimated that, by 2030 TC, would become the fourth leading cancer diagnosis, following breast, prostate and lung carcinomas [2]. We are still lacking consensus regarding the cause of this phenomenon. However, in our opinion, the major problem is not related to TC growing incidence itself, but rather whether we are ready for it. Another, not less important issue according to Heller is that: “We may be diagnosing and treating cancers that have no clinical significance” [3]. Thus, the distinction between patients who do not need to be treated from those with poor prognosis is an important issue that needs to be resolved in the nearest future.

TC usually is an indolent disease, characterized by good outcomes. Therefore, an adequate management is particularly important to avoid the overdiagnosis and overtreatment in a low-risk group on the one hand and on the other hand the underdiagnosis and undertreatment of high-risk patients. This idea has been reflected in the current, recently published, evidence-based American Thyroid Association (ATA) guidelines for differentiated thyroid cancer (DTC) [4]. Nevertheless, there are still many doubts, which have become a platform for an extensive discussion between American and European scientists carried out during the Berliner meeting, organized by the European Association of Nuclear Medicine (EANM), held in June 2016. Some of ATA recommendations were considered to be controversial from the European point of view, among others the indications for radioiodine (RAI) ablation in DTC, which was addressed in two papers published by a German group [5,6].

In this article we mainly focus on the current advantages in DTC management. We discuss a preoperative molecular diagnostics, new findings concerning histopathological examination, as well as some controversies regarding DTC management, particularly the extent of surgery and adjuvant radioiodine (RAI) therapy, and new treatment modalities for RAI-refractory DTC (RR-DTC). Nevertheless, we decided to provide some information referred to medullary thyroid cancer (MTC), its clinical management based on histopathology and *RET* (ret proto-oncogene) mutation genotype, follow-up with a special focus on serum calcitonin as an important marker, and targeted therapy applied in advanced MTC. In addition, we paid our attention to anaplastic thyroid cancer (ATC), a highly lethal neoplasm, leading to death in nearly 100% of patients due to the lack of effective treatment options.

## 2. Follicular-Cell Derived Thyroid Cancers

### 2.1. Is the Increase in TC Incidence a Real Phenomenon or an Artifact Overdiagnosis?

During the last several decades a dramatic increase in a number of patients diagnosed with TC has been observed worldwide. Although the mortality rates for TC remain rather stable and low, the rise in its incidence represents the highest rate comparing to any other malignant neoplasm. In the US the number of TC cases for both women and men has nearly tripled since 1975 by an increase from 4.9 cases per 100,000 persons up to 14.3 cases per 100,000 persons in 2014 with the mortality rate of about 0.5 per 100,000 persons per year [7]. One may say that we are dealing with an epidemic rise, or being more specific, an epidemic of TC diagnosis. This phenomenon is mainly related to the growing incidence of papillary TC (PTC) [8]. It is still the matter of debate whether it is a consequence of improved diagnostic methods and screening or represents a real change in TC incidence due to some environmental factors [9,10,11]. It is very probable that more accurate and easy accessible imaging examinations result in a better detection of thyroid tumors. Palpable nodules are estimated to be present in 3% up to 7% of the world population, and this frequency may reach even 76% when ultrasonography is used [12]. Incidental thyroid nodules (ITNs) are also seen in up to 25% of contrast-enhanced chest CT scans, 16–18% of neck CT and MRI, and 1–2% of Fluorodeoxyglucose (18FDG)-PET scans [13]. Approximately 5% of all ITNs appear to be malignant. However, the malignancy rate of ITNs depends on patients’ selection or the detection technique. This rate, reaching 33% to 35%, is highest in lesions found in FDG-PET, whereas the malignancy rate in ITNs revealed by CT and MRI ranges from 0% to 11% [13]. Most TCs are small PTCs, which usually show a slow growth and could not be diagnosed without imaging studies. It has been demonstrated that patients, who died due to other causes frequently present an occult PTC. In Finnish population this frequency was about 35.6%, which suggested that occult PTC was not only a very common lesion but also clinically insignificant in many cases [14]. This increased detection of thyroid nodules might have resulted in TC overdiagnosis. An analysis based on SEER data including the years between 1998 and 2010 revealed that 5- and 10-year survival of patients who underwent total thyroidectomy for papillary microcarcinoma (PTMC) was similar to that of a general population [15]. Moreover, an increasing number of ITNs was classified as “follicular variant of PTC” (FVPTC) rather than follicular adenomas (FA) [11], which in consequence could be responsible for the number of overdiagnosed and ovetreated TC cases. This has been changed since the reclassification of encapsulated noninvasive follicular variants of PTC as a benign lesion, currently renamed as “noninvasive follicular thyroid neoplasm with papillary like nuclear features” (NIFTP) [16].

Most arguments speak for more accurate detection methods and their global use as a cause of growing number of TC cases. However, Chen et al. believed that only half of them could be explained by “overdiagnosis”, whereas the remaining half probably reflected a true increase in TC incidence rate [17]. The authors indicated the bigger number of all thyroid tumors regardless of their size suggesting other explanations than a better diagnostic scrutiny. This idea was confirmed by Vergamini et al., who demonstrated an increase of DTC incidence also among children, adolescents and young adults in the US with growing trends for larger tumors (over 2 cm) [18]. Therefore, environmental factors, lifestyle impact, concomitant diseases (e.g., chronic inflammation) as well as their coexistence should be considered as the possible causes of TC epidemic. One of the well-known risk factors of TC development is a low-dose radiation exposure in childhood because the thyroid is a very radiosensitive organ at a young age. A significant increase in childhood TC was reported after the accident in Chernobyl nuclear power plant in 1986. Similarly, medical diagnostics using X-rays, CT scans and RAI therapy may influence the risk of TC. However, the decrease in *RET/PTC* rearrangements, assumed to be a molecular fingerprint of radiation-induced PTC, has been observed over the time, which speaks against the radiation exposure as a reason of TC epidemic [8,19]. The other authors pointed a growing prevalence of *BRAF* V600E (B-Raf proto-oncogene, serine/threonine kinase) mutation in classical PTC variant. This phenomenon was observed in different geographical localizations [19,20,21,22], so common or very similar factors, perhaps dietary or chemical ones, might be responsible for this trend. It is well known that higher iodine intake is associated with a higher PTC incidence and the addition of iodine to dietary salt, introduced in the US in the 1920s, may be associated with a higher PTC prevalence and a decrease of the frequency of follicular thyroid carcinoma (FTC). A recent Chinese study reported a two-fold higher risk of *BRAF* V600E mutation in regions with high iodine levels in drinking water comparing to those of normal iodine concentration [23]. Higher incidence of *BRAF*-positive PTCs has also been observed in a volcanic region of Sicily with high concentrations of boron, vanadium, manganese and iron in drinking water and, what is interesting, the highest increase of TC cases in the US was noticed on volcanic islands of Hawaii [19]. However, considering the latter localization, an increased iodine intake may not be excluded. The increased frequency in *RAS* (proto-oncogene, GTPase) mutations was also reported, but within FVPTC. These alterations are mainly associated with exposure to chemical carcinogens, as demonstrated in hepatocellular carcinomas positive for *RAS* mutations in workers exposed to vinyl chloride [24], or in lung adenocarcinomas associated with tobacco smoking [25]. Among chemical factors involved in TC increasing rate are polybrominated diphenyl etherflame retardants (PBDEs). It has been reported that PBDEs lead to thyroid dysfunction and the exposure to these chemicals in the US has increased significantly over the last few decades. However, the question whether PBDEs are responsible for TC needs further investigation [26].

Some data demonstrated an increased TC risk in obese individuals [27,28]. However, considering the relationship between diabetes, a frequent consequence of obesity, and TC, the data are inconsistent and rather indicate the lack of a possible association. Another important issue is chronic autoimmune Hashimoto’s thyroiditis (HT) showing a growing incidence, parallel to TC. It seems to be particularly important since HT patients demonstrate a chance of TC development three times greater than normal population [29]. It may suggest a strong link between chronic inflammation and cancer.

As TC is more frequent in women also the role of estrogen, as a possible risk factor, has to be analyzed, especially considering a higher estrogen exposure mainly due to its medical use (hormone replacement therapy and contraception). Estrogen and its receptors were important factors influencing proliferation, migration, and invasion of TC cells in in vitro studies [30,31]. However, such effects have not been unequivocally demonstrated in humans and further data are needed [11]. A comparison between 40 TC women and 40 age-matched controls showed that estrogen metabolism was unbalanced in TC and suggested a possible role of estrogen-DNA adducts in TC initiation [32]. While, in another multicenter, prospective cohort study a significantly higher TC risk was observed in postmenopausal women with hysterectomy comparing to women without hysterectomy, regardless of ovarian status. Interestingly, when a hysterectomy group was considered alone hormone therapy was related to a lower TC risk. This report does not support the hypothesis that exogenous estrogen may promote TC development [33]. As the results of published studies in women are divergent, so far we are not able to unequivocally assess the role of estrogen in TC development.

### 2.2. Is the Diagnosis of Thyroid Cancer an Unquestionable Statement (NIFTP, FTC vs. FA)?

Follicular variant of PTC has two different subtypes: encapsulated and infiltrative (or non-encapsulated). Encapsulated FVPTC (EFVPTC) is a controversial diagnosis in thyroid pathology [34]. Recently published studies have proved that EFVPTC displays an indolent behavior. The name “noninvasive follicular thyroid neoplasm with papillary-like nuclear features” was based on the results of clinical observation of 109 EFVPTC patients with the median recurrence-free survival of 13 years [16]. Sixty-seven patients were treated only with lobectomy, without RAI ablation. The cases were re-evaluated by an international group of expert pathologists to establish standardized diagnostic criteria. The use of a new term NIFTP allows avoiding the label “cancer” for such very low-risk tumors. According to the published data, there were only two cases (0.6%) with disease recurrence among 352 well documented noninvasive encapsulated/well circumscribed FVPTC [16].

Pathological criteria for NIFTP diagnosis are strict. The main inclusion criteria for NIFTP focus on tumor capsule (present without invasion) or clear tumor demarcation, follicular growth pattern and nuclear features of PTC at least focally present [35]. The nuclear PTC features involve enlargement, crowding or overlapping, elongation, irregular contours, grooves, pseudoinclusions, and chromatin clearing. Among the exclusion criteria for NIFTP diagnosis are tumor capsular invasion, vascular invasion or tumor infiltration, solid/trabecular or insular growth in more than 30% of tumor, even one well-formed papillary structure, psammmoma bodies, tall cell, columnar or cribriform morular morphology, necrosis (not after fine needle biopsy), and mitotic index >3 mitoses/10 HPF [16,36]. Importantly, the newly defined, even stricter criterion for NIFTP, published on the website of the College of American Pathologists in June 2017, is that the presence of any papillary structure excludes NIFTP diagnosis [36]. NIFTPs may harbor *RAS* gene mutations, *PAX8/PPARG* translocations and *THADA* fusions [16].

Due to so strict pathological criteria, a high quality of tissue fixation, gross examination and tissue processing is required to diagnose NIFTP. The entire capsule of a lesion or tumor normal interface must be embedded for the evaluation of microscopic invasion. Suboptimal tissue fixation or processing may result in artifactual nuclear features similar to PTC. The diagnosis of NIFTP tumors should not be attempted on frozen sections [37]. Frozen section is not an optimal examination for evaluating inclusion and exclusion criteria for NIFTP because PTC nuclear features, the entire capsule of the lesion, and invasion of the surrounding thyroid cannot be adequately assessed due to a smaller amount of tissue examined and lower quality of slides, in comparison to paraffin embedded slides.

Current data allow to diagnose NIFTP in tumors more than 1 cm and less than 4 cm in diameter. To date, there has been no study with a long term follow up addressing the outcome of tumors smaller than 1 cm in diameter diagnosed with NIFTP [38]. Only a preliminary international study of 48 patients with PMTC that behave similarly to NIFTP has been published [39]. Another international study of large (more than 4 cm) noninvasive FVPTC showed that such tumors can be safely and adequately dealt with surgical treatment alone and diagnosis of NIFTP may be implemented [40]. Similarly, there are no reports showing that noninvasive FVPTC with oncocytic features has indolent clinical outcome as NIFTP without oncocytic features. Future studies concerning these tumors are needed [38].

It has to be emphasized that the diagnosis of NIFTP is impossible on the basis on a cytological material coming from fine needle aspiration biopsy (FNAB). There are no cytological criteria for recognizing NIFTP. Strict histopathological criteria described above are evaluable only in a post-operative material.

The frequency of EFVPTC diagnosis has increased in the last decades. EFVPTC represents 10–20% of all TCs that have been recently diagnosed in Europe and in the US [41]. The literature does not comprise relevant data concerning the frequency of newly established entity “NIFTP” fulfilling the strict diagnosis criteria presented above. It can be expected that NIFTP frequency will be lower than frequency of EFVPTC. Newly strict criteria of NIFTP, given above, are undergoing a dynamic evaluation process and will be able to show the real frequency of this entity soon.

One may attempt to look for an analogy between NIFTP and FA as far as between invasive FVPTC and encapsulated FTC (EFC). The similarity in the clinical course between NIFTP and FA was reported by Ganly et al. [42]. Considering invasive encapsulated FVPTC and EFC there were no differences in the rate of lymph node metastases and the degree of vascular or capsular invasion. Nevertheless, it was emphasized that capsular and/or vascular invasion more than nucleus features was related to poor behavior of encapsulated follicular tumors [42]. Therefore, a very careful evaluation of capsular and vascular invasion in encapsulated tumors using additional levels of blocks or immunohistochemistry for decorating vessels is required. The proper and balanced assessment of the microscopic criteria allows for conducting an appropriate tumor classification and avoidance of patient’s overtreatment.

### 2.3. What Is the Role of Gene Expression/Mutation Classifier in the Diagnosis of TC?

The decision on which thyroid nodule needs surgery and which does not is still challenging for clinicians. FNAB is the most effective method for screening and diagnostics of ITNs. However, up to 30% of biopsies are considered as indeterminate by a cytological examination [43]. According to Bethesda System for Reporting Thyroid Cytopathology, there are three categories within the indeterminate group: “atypia of undetermined significance (AUS)/follicular lesion of undetermined significance (FLUS)”, “follicular neoplasm or suspicious for follicular neoplasm (FN/SFN)”, and “suspicious for malignancy (SUSP)” with the frequency among thyroid lesions of 2%–18%, 2%–25% and 1%–6%, respectively [4]. According to a multicenter study of Wang et al. about 74% of tumors diagnosed as indeterminate at initial FNAB undergo surgery [44]. However, more than two-thirds of these cases are postoperatively benign.

To decrease the number of overtreated indolent thyroid tumors, novel molecular tests are being developed to help in distinguishing between benign and malignant lesions. These tests may be based on alterations observed at the level of a gene, protein or miRNA expression. Generally, classifiers using either immunohistochemical examinations of different markers combinations, based on miRNA, or alterations in gene signatures are characterized by a high sensitivity and a negative predictive value (NPV). These tools are considered as “rule out” tests. As opposed to them, diagnostic tools that use genetic markers known from their ability to induce TC, like genetic mutations or rearrangements, show a high specificity and a positive predictive value (PPV) and these are aimed at sensitive detection of malignancy (“rule in” tests) [45].

There are several commercial tests available in the diagnostics of thyroid nodules, among them Afirma Gene Expression Classifier (GEC) (Veracyte Inc., South San Francisco, CA, USA) and ThyroSeq (commercially offered by CBLPath, Rye Brook, NY, USA) (Table 1). The first one, GEC classifier, measures the expression of 167 gene transcripts, and is based on two gene panels: one consisting of 142 genes, which expression changes enable to distinguish benign and malignant thyroid lesions, and another one with six “cassettes” including 25 genes, which enable screening analysis for less frequent thyroid lesions, such as MTC, oncocytic follicular lesions or melanoma, breast or kidney cancer metastases [46,47]. The Afirma GEC was trained against the known histopathological and cytopathological thyroid samples and tested against unknown samples with NPV and estimated specificity of 96% and 84%, respectively [46]. The test was validated in a blinded prospective multicenter trial, in which a high NPV of 95% and 94% was obtained for AUS/FLUS and FN/SFN categories, respectively [47]. The risk of malignancy was 5% and 6% within the respective Bethesda classes in the case of a “benign” GEC result. This study showed that GEC is a weaker tool for Bethesda category V (SUSP), since NPV was only 85%, and the risk of malignancy with a “benign” GEC result was 15%. Although “benign” result significantly decreases the number of unnecessary surgeries, the “suspicious” result is less precisely defined. Thanks to the use of GEC in a daily clinical practice the recommendation for surgery in patients with indeterminate biopsy decreased by 93%, although only 44% of tumors with a “suspicious” GEC result appeared to be malignant postoperatively [48]. This tendency was confirmed in further studies also demonstrating high NPV and sensitivity rates. However, PPV rates were low and ranged between 14% and 57% [49]. It has to be noted that for each diagnostic test the values of PPV and NPV depend on the cancer incidence in a studied group. For Afirma test this prevalence of malignancy in the indeterminate thyroid lesions was between 15% and 21%. Outside this range the Afirma test provided less informative results with moderate influence on treatment decisions [50]. The knowledge of TC frequency in different Bethesda categories, in a particular population seems to be critical for the proper interpretation of GEC results. Another GEC limitation is the fact that most benign Hürthle-cell nodules are classified by the test as “suspicious” [43,51]. Additional challenge for the Afirma GEC is NIFTP, which at the moment of GEC development was classified as malignant lesion and this standard was used to train and verify the test. Currently, these lesions are considered as benign ones. According to Lastra et al. FVPTCs accounted 73% of cases of category III and IV according to Bethesda System, classified as GEC “suspicious” [43]. Overall negative result of the GEC led to decrease of the number of thyroidectomies from 74% to 7.6% within the group of patients with indeterminate nodules [50].

An example of “rule in” test is ThyroSeq. It is a NGS-based gene mutation and fusion test identifying more than 1000 mutations located within 14 genes, 42 fusions and analyzing expression of eight genes [52,53]. The performance assessment of ThyroSeq v2 test in retrospective and prospective cohorts by Nikiforov et al. resulted in overall 90% sensitivity, 93% specificity, 96% NPV, 83% PPV, and accuracy of 92% [53]. These results may suggest that this molecular test has both “rule in” and “rule out” properties. However, similar to GEC, the ThyroSeq v2 test predictive values correlate with a pretest risk of malignancy. With the risk between 5% and 15% for AUS/FLUS, ThyroSeq v2 would be expected to have still a high NPV, between 98% and 99%, but PPV would decrease up to 40–69%. Low pretest risk of malignancy is related to a low PPV and makes the test not satisfactory as a “rule in” tool.

There are also other molecular tests, such as ThyGenX (Interpace Diagnostics, Parsippany, NJ, USA), dedicated for Bethesda III and IV category and enabling identification of more than 100 mutations of eight genes related to TC, or ThyroMIR (Interpace Diagnostics, Parsippany, NJ, USA), suggested for patients with a negative result of the ThyGenX.

All these mentioned tests may help in a better and more accurate diagnostics of TC. However, they are very expensive and influenced by many pre-analytic factors. One has to be aware that the reclassification of EFVPTC as a benign tumor leads to decrease of PPV for all available molecular tests.

### 2.4. What Is the Tumor Size That Justifies Total Thyroidectomy?

The optimal extent of surgery in DTCs has been widely discussed for many years [54]. Regardless of the impact of different prognostic factors, the supporters of total thyroidectomy always believe that such approach reduces the risk of recurrence, increases the effectiveness of RAI therapy, allows the use of thyroglobulin as a sensitive marker and eliminates the possibility of tumor dedifferentiation. On the other hand, the opponents propose the less extensive surgery in low-risk patients. Criticism of a radical surgical strategy in DTCs is based on the following premises: generally benign course of PTC and minimally invasive FTC; the lack of the necessity of RAI ablation after less extensive surgery; and the lower risk of complications [55,56,57,58,59,60].

The controversy concerning the extent of surgery results from the retrospective nature of available analyses, heterogeneity of patient groups, distinct diagnostic criteria in the assessment of recurrence risk, and too short postoperative follow-up. A prospective, randomized study that would allow a comparative assessment of the outcomes in patients after total and less than total thyroidectomy has not been performed yet probably due to the requirement of a long-term follow-up [61].

The problem is of a paramount importance due to the increasing number of low-stage TCs diagnosed preoperatively. According to the 2009 ATA recommendations, unifocal PTC ≤1 cm in diameter without the presence of clinically or radiologically suspicious lymph nodes was considered as the indication for lobectomy [62]. Adam et al. compared treatment results in patients with PTMC, who underwent lobectomy and total thyroidectomy. They did not observe any differences in the prognosis between the groups [63]. Analyzing our own material, we retrospectively evaluated a group of 1235 DTC patients treated or diagnosed between 1986 and 1998. We demonstrated that the extent of surgical treatment was a significant independent prognostic factor of recurrence for tumors >1 cm, which was consistent with other reports [64]. Our non-randomized prospective clinical trial, carried out between 2011 and 2015, confirmed that lobectomy is acceptable in patients with a clinical advancement of cT1aN0M0 [65].

The primary tumor size justifying lobectomy was changed by the newest ATA Guidelines [4]. Currently, the cut-off value that constitutes the limit for lobectomy is ≤4 cm, if there is no infiltration of thyroid capsule and/or surrounding organs, no distant or locoregional lymph nodes metastases, and no history of radiotherapy of the head and the neck (Table 2). Such management is also recommended by the National Comprehensive Cancer Network (NCCN) and the British Thyroid Association [66,67]. The published, retrospective data demonstrated no differences in the distant outcomes for intrathyroidal tumors ≤4 cm, regardless of surgical approach (lobectomy vs. thyroidectomy) [68,69]. However, the results of these studies are not consistent with other reports. Bilmoria et al. analyzed a group of 50,173 patients, with a mean follow-up of 70 months and demonstrated that for tumors ≥1 cm, lobectomy resulted in a higher risk of recurrence and death [70].

It has to be stressed that not the tumor diameter itself defines the limit for thyroid surgery [61]. If any of the above-mentioned risk factors are present, routine total thyroidectomy is still recommended. Patient’s preference is also important [4]. One should remember that many risk factors crucial for prognosis are known only postoperatively, and subsequent reoperation might be necessary [71].

Abandoning primary total thyroidectomy in DTCs with the tumor diameter of >1 cm and ≤4 cm is not commonly accepted and requires further evaluation in prospective studies. In the US a single lesion limited to the thyroid gland is more frequently observed than in some European countries with a high prevalence of multinodular goiter. Therefore, it is easier to implement the procedure limited to lobectomy. In cases of multinodular goiter and autoimmune thyroiditis the indications for total thyroidectomy, even in case of small-diameter cancers, are wider. It seems that, currently, it is possible to accept the tumor diameter of 1 cm for unifocal PTC without other risk factors as a limit of possible abandonment of primary and secondary total thyroidectomy [72]. Additionally, some studies are conducted to completely replace surgical treatment with patient surveillance in low DTC stages [71].

### 2.5. When Is Adjuvant Radioiodine Therapy Necessary?

The role of adjuvant RAI treatment in DTC has been extensively discussed during the recent years. In particular, the indications for postoperative RAI therapy in low-risk DTC are controversial due to the lack of unequivocal proofs of its effectiveness, as the data coming from retrospective analyses are divergent. These issues are reflected in the current ATA guidelines, which classify the indications for postoperative RAI administration in the three following categories: therapy (remaining DTC tissue is present), adjuvant therapy (for high-risk subjects), and thyroid remnant ablation. RAI ablation is definitely not recommended in PMTC. It is not routinely recommended in other patients initially classified as ATA low-risk. However, this is a weak recommendation and ATA members assessed the quality of evidences as low. On the other hand, RAI adjuvant therapy is strongly recommended for ATA high-risk group based on moderate-quality evidences. The decision regarding RAI administration in ATA intermediate-risk group is equivocal. RAI adjuvant therapy just should be considered [4]. Such approach allows for an individual decision in each DTC intermediate-risk patient, depending on patient and physician preference. Therefore, the position presented in a recently published review [73] seems to be a bit conservative in the light of the current ATA guidelines. The American guidelines clearly limited the role of RAI complementary therapy in DTC treatment. Such approach raises some doubts in different European centers. According to German authors, “when it comes to nuclear medicine the document (ATA GL) appears to lack both knowledge and substance, resulting in uncertainty and ambiguity” [6]. Therefore, EANM refused to endorse them [5]. In addition, Polish guidelines are less restrictive regarding post-operative RAI therapy [74]. The only way to resolve the question of whether RAI has to be administered in low-risk DTC patients is via prospective studies. Currently, two of them are recruiting participants: IoN study in the United Kingdom (NCT01398085) and Estimabl 2 study in France (NCT01837745).

### 2.6. Are There Any Molecular Factors That Determine TC Aggressiveness?

Considering the fact that most TCs are indolent tumors, it is of high importance to be able to recognize, which of them evolve to an aggressive form. This is crucial for an adequate approach and to preserve patients from unnecessary procedures and overtreatment. Until recently, it has been thought that clinical and histopathological factors are much more important in TC diagnosis and risk stratification than genetic ones. However, each subsequent publication on TC molecular aspects provided the evidences on a significance of genetic alterations not only in the tumorigenesis but also in TC aggressiveness (Table 3). The Cancer Genome Atlas (TCGA) study, focused on genetic, epigenetic and proteomic background of PTCs, classified PTCs into two main molecular types, named by the authors as RAS-like and BRAF-like [75]. The latter one consisted mainly of PTCs harboring *BRAF* V600E mutation and represented a very heterogeneous group. This fact may support an inconsistence noticed, when the association of this alteration and poor prognosis is considered. Although *BRAF* V600E mutation is the most frequent genetic alteration in PTC, present in 36% to 83% of cases [76], its usefulness as a prognostic factor is questionable. Most studies indicated a significant association between *BRAF* mutation and other factors of poor prognosis such as older age, male gender, higher tumor staging, tumor size, extrathyroidal extension, lymph node metastases, even distant metastases, and recurrence [77,78,79,80,81,82]. However, the other reports did not confirm above-mentioned associations [83]. *BRAF* V600E mutation was associated with a decrease in expression of several thyroid-specific genes, which may be responsible for the loss of RAI avidity in recurrent PTC [84]. Noteworthy, childhood PTC, positive for *BRAF* V600E mutation, does not show a more aggressive clinical course [85]. More interesting, Geng et al. presented a negative correlation between the mutation and aggressive clinicopathological features of pediatric PTC [86]. The data concerning prognostic value of the *BRAF* mutation, its impact on DTC recurrence rate and mortality were summarized by Xing in a few recently published studies [87,88,89,90].

The aspect of *BRAF* V600E mutation clonality remains unresolved. It is crucial to answer the question, whether we are dealing with clonal or sub-clonal alteration since it may dramatically change the understanding of PTC molecular background and influence clinical approaches, including risk stratification. Data obtained by some authors, including TCGA study indicated on a clonal nature of the *BRAF* V600E mutation, demonstrating its homogeneous distribution across the tumor [75,91,92]. Sub-clonal nature of this genetic alteration was observed first by Guerra et al., who used pyrosequencing and provided the exact percentage of alleles with the mutation [93]. The results were confirmed by further studies showing that *BRAF* V600E mutation was rather a rare clonal event in primary PTC tumors [94,95]. Based on previous reports the medical community agreed that this particular *BRAF* mutation, although very promising at the beginning, could not be used as a single marker of PTC aggressiveness. It may be considered only in a multivariable context, combined with other prognostic factors.

Recently, *TERT* promoter mutations have been discovered in melanomas [96]. The same year, in 2013, Liu et al. reported the presence of these alterations in follicular cell-derived TCs with two hotspot positions, located at −124 and −146 bp upstream from the ATG start site (chr5: 1,295,228 C > T named as C228T and 1,295,250 C > T named as C250T respectively) [97]. The frequency of these mutations, with the first one (C228T) being more common, is highest in poorly differentiated (PDTC) and ATC with the rates of 29% and 33%, respectively, while, considering DTC, these rates decrease up to 17.1% in FTC and 7.5% in PTC [98]. In several studies, the relationship between the *TERT* promoter mutations and clinicopathological features, like older age at diagnosis, larger tumor size, higher disease stage, and distant metastases as well as clinical outcomes was demonstrated [97,98]. What is important, most *TERT*-positive PTCs harbor *BRAF* V600E mutation as well. It has been suggested that *TERT* promoter mutations but not the *BRAF* alteration are responsible for poor prognosis. However, a series of studies showed that prognostic effects related to *TERT* promoter mutations were visible only when analyzed with *BRAF* mutation and they decreased or disappeared, when mutations occurred separately [99,100,101]. These data suggest that co-existence of these two molecular events is mandatory for promoting tumor aggressiveness. A previous study of Vinagre et al. supported this hypothesis [102]. The authors observed that *TERT* mRNA expression was higher in PTCs positive for *TERT* promoter mutation and *BRAF* V600E mutation comparing to PTCs with only one of these alterations [102]. Therefore the association between *TERT* promoter and *BRAF* mutations could be considered as a unique hallmark of a subgroup of DTC patients with aggressive form of the disease and poor outcome. There are also data pointing similar interaction between *TERT* promoter alterations and *RAS* somatic mutations. Song et al. observed that these two molecular events, when occurring together, led to the worsening of clinic-pathological features and overall outcome of DTC patients [101]. The prognostic significance of *RAS* mutations alone is controversial since they are present also in benign thyroid lesions. Although the frequency of *RAS* mutations is higher in more aggressive TC types such as PDTC (55%) and ATC (52%) and there is an association between *RAS* mutations and the presence of distant metastases in PDTC, the prognostic value of these genetic alterations is not clear [76].

*TP53* inactivating mutations are considered to be a hallmark of ATC due to their high frequency in this type of TC. They are observed in up to 59% of ATC cases. Considering other TC types *TP53* mutations are present in 10% of PDTC patients [103] and rarely in well-differentiated TCs. In the recent TCGA study barely 0.7% of PTCs carried such mutations [75]. However, Nikiforova et al. demonstrated the alterations in the *TP53* gene and/or p53 protein expression in a small proportion of aggressive PTCs and FTCs [52]. There are no doubts that *TP53* inactivating mutations are a late event, occurring during TC progression. The loss of p53 probably leads to deregulation of apoptotic pathways, genomic instability, and, as a consequence, acquisition of additional mutations and progression of TC to aggressive forms. As hallmarks of ATC also mutations in genes of WNT signaling pathway, *CTNNB1* (beta-catenin), *APC* and *AXIN1*, were reported due to their high prevalence in ATC (62% to 82% of cases) [104,105,106]. However, the other studies based on next generation sequencing, observed WNT pathway mutations in only 3% to 4.5% of ATCs [107,108].

Activation of the phosphatidylinositol 3-kinase (PI3K) signaling pathway seems to be an important event in TCs development with mutations of *PTEN* and *PIK3CA* being most frequent in advanced TCs, mostly in ATC [103]. The alterations of the PI3K pathway are more common in FTC comparing to PTC. Moreover, as demonstrated in PDTC, these genetic and epigenetic modifications usually coexist with alterations of the mitogen-activated protein kinase (MAPK) pathway (RAS/RAF/ERK activating mutations) suggesting the role of PI3K pathway as the second hit in thyroid carcinogenesis [109].

Recently, miRNAs have demonstrated their role in TC progression and as potential markers of malignant thyroid tumors. An increased aberrant expression of some miRNA, mainly miR-222, miR-221, and miR-146b in PTCs, comparing to normal thyroids was reported, with the last one significantly associated with aggressive form of BRAF-positive PTCs [110]. Moreover, up-regulated expression of miR-146b was present mainly in PTCs (89%) and FVPTCs (41%), but not in FTCs, ATCs, PDTCs, or FAs (7%, 8%, 0% and 0%, respectively) [111]. However, the measurement of the miR-146b levels in pre-operative material needs further studies.

### 2.7. Could Small Thyroid Carcinomas Be Managed without Surgery by Watchful Waiting Strategy?

As it was mentioned above PTMC was frequently diagnosed (5.6–35.6%) at autopsy in patients, who died due to different causes [14,56,112]. Therefore the “watchful waiting” strategy, proposed in the management of prostate cancer [113,114], might be considered in PMTC. Thus far, such approach has been accepted only in Japan. In 1993, an active surveillance trial was initiated in Kuma Hospital in Kobe [115]. Two years later, a similar study began in the Cancer Institute Hospital in Tokyo [116]. The groups, enrolled in the surveillance trials, were gradually extended and the results were reanalyzed over the time [117,118,119,120]. During 10-year follow-up, an increase in the tumor size by at least 3 mm was observed in 8%, whereas locoregional lymph node metastases were diagnosed in 3.8% of 1235 PTMC Kobe patients, without a negative impact of the final outcome. Those patients who underwent surgical treatment demonstrated no other events during the further follow-up [71]. Similar results were obtained in Tokyo. In a group of 230 patients during a 10-year follow-up a significant increase in tumor size was observed in 7% of patients and lymph node metastasis in 1%. Delayed surgical procedure did not influence the overall survival [71]. Summarizing the results of these studies, Ito et al. [71] observed that “watch and wait” strategy was safe, if patients were properly selected. The vast majority of patients did not undergo any surgical procedure during the long-term follow-up.

Except for obvious cases (e.g., metastatic or suspicious lymph nodes, infiltration of the surrounding structures, and cervical radiotherapy in the past), these authors recommended surgical treatment for patients with the unfavorable tumor location, which increased the risk of laryngeal recurrent nerve infiltration during progression. Additionally, the unfavorable outcomes were less frequently observed in the elderly. Therefore, observational management is the treatment of choice in this group of patients. Despite a more frequent occurrence of lymph node metastasis in younger patients, Japanese authors believed that age was not a factor excluding young patients from the surveillance trial. The risk of lymph node metastases was the same and did not depend on whether patients were subjected to “watch and wait” or underwent primary thyroidectomy [71].

It is well known that PTMC is related to an excellent prognosis. Nevertheless, in some patients the unfavorable course of the disease (distant metastasis, cancer-related death) was observed [121,122,123]. However, no reliable clinical or molecular factors allow predicting the unfavorable outcome of the disease. According to the ATA guidelines, further studies are necessary to select patients, who can be safely monitored. Additionally, an algorithm for their further follow-up should be clearly defined including the frequency of follow-up visits, the significance of thyroglobulin assays, and indications for surgical intervention [4]. According to Brito et al., PTMC patients considered for “watch and wait” approach have to undergo a careful evaluation including: tumor/neck ultrasound characteristics (e.g., size of the primary tumor and the location of the tumor within the thyroid gland), patient characteristics (e.g., age, comorbidities, and willingness to accept observation), and medical team characteristics (e.g., availability and experience of the multidisciplinary team) [124]. It was demonstrated that the best candidates for such strategy are patients over 60 years of age with a single intrathyroidal nodule with well-defined borders, who are willing to accept an active surveillance management approach [124].

Considering the increasing number of PTMC patients as well as a relatively indolent nature of the disease, the “watchful waiting” strategy, in which active treatment is delayed until the signs of clinical progression, seems to be a rational option for a selected group of patients [125]. An open-label, non-randomized clinical study to evaluate the outcomes of an active surveillance PMTC is currently recruiting the patients in the US (NCT02609685).

### 2.8. Is There a Molecular Phenotype of Indolent Thyroid Cancer?

Recent studies indicated that some genetic alterations might be used as markers of TCs aggressiveness (Table 3). On the other the data concerning molecular phenotype of indolent TCs are scarce. Torregrossa et al. suggested that rare *BRAF* mutations, localized within the exon 15, may represent a pre-operative predictive factor of good prognosis [126]. The authors found rare *BRAF* alterations, with K601E mutation being most frequent, in 1.8% of PTC cases, mostly in FVPTCs and PTMCs (with follicular pattern) as well as in a few cases of classical PTC variant (CVPTCs), and in one PTC case with trabecular-solid pattern of growth (TSVPTC). PTCs carrying rare *BRAF* mutations and *BRAF* wild type tumors were very similar regarding their aggressiveness. The prevalence of these alterations in FVPTCs, support their consideration as low-risk tumors, especially when they are encapsulated. In other study tumors with *BRAF* K601E alteration presented less aggressive behavior comparing to *BRAF* V600E-positive ones and were classified as RAS-like PTCs [75]. These rare *BRAF* alterations were not described in PDTC and ATC [107]. These data may support the idea of their role as markers of indolent TCs. However, further analyses are required. Some authors suggested a possible association between *RAS* mutations and low-risk TCs, especially FVPTCs with favorable clinical and sonographical profile. Kakarmath et al. observed that *RAS*-positive malignancies were usually cytologically indeterminate nodules and did not present suspicious sonographic features [127]. Moreover, considering older patients *RAS*-positive TCs were generally larger in size comparing to *BRAF* V600E-positive TCs. The authors of this observation suggested that *RAS* mutations stimulated clonal growth of follicular thyroid cells, however, a “second hit” was necessary for transformation into an aggressive form. Similar data were obtained by Medici et al., who additionally demonstrated that the type of *RAS* mutation was also important. The thyroid nodules with *KRAS* mutations were associated with a lower risk of TC comparing to nodules with *HRAS* and *NRAS* alterations [128]. However, the usage of *RAS* mutations as markers of indolent TC is controversial since they have also been found in very aggressive PDTC and ATC. They may be considered only with clinical-sonographic-pathological profile of certain thyroid nodule.

## 3. A Place for Multi Kinase Inhibitor (MKI) Therapy in RAI-refractory DTC (RR-DTC)

Until recently, the available therapeutic options for advanced RR-DTC were limited to radiotherapy, local treatment modalities or chemotherapy, without any promising outcomes [4]. New possibilities opened up along the discovery of different tyrosine kinases and their role in TC pathogenesis. Multi Kinase Inhibitors (MKIs) are a new group of drugs with the activity against receptors of different growth factors leading to the inhibition of tumor cells growth and proliferation. Despite of numerous clinical trials evaluating different drugs [129] only two MKIs unequivocally demonstrated their effectiveness in RR-DTC in randomized, placebo-controlled, phase III clinical studies: sorafenib [130] and lenvatinib [131]. Sorafenib, in comparison to placebo, significantly prolonged progression-free survival (PFS) in RR-DTC patients, 10.8 months versus 5.8 months, respectively [130]. Similarly, the median PFS in RR-DTC patients receiving lenvatinib was significantly longer than in placebo group 18.3 months versus 3.6 months, respectively [131]. Regardless of their beneficial impact on PFS neither sorafenib [130,132] nor lenvatinib [131] prolonged the overall survival (OS) in RR-DTC. Nevertheless, considering the impact of both MKIs on OS one should remember that upon progression, patients who received placebo were allowed to start an active treatment under open-label phase. Only a subgroup of patients above 65 years of age, who received lenvatinib, showed a significantly longer OS comparing to placebo [133].

As a consequence of their pathomechanism of action MKIs do not constitute a curative therapy. The drugs inhibit tumor cell growth and proliferation, but do not kill cancer cells. Therefore the treatment is life-long and rapid cancer progression may be expected after drug withdrawal. On the other hand MKIs administration is related to numerous side effects in nearly all treated patients, which rarely may be severe, life threatening or even cause a death [134]. Therefore, according to the current ATA guidelines MKIs therapy may be considered “with regard not only to potential benefits but also about potential side effects and risk of therapy and alternative therapeutic approaches including best supportive care” [4]. “Imminently treating disease progression expected to require intervention and/or to produce morbidity or mortality < 6 months; symptomatic disease not adequately addressable using directed therapy; or diffuse disease progression” are the only indications for MKIs administration [4]. While, the presence of “active or recent intestinal disease, liver disease, recent bleed or coagulopathy, recent cardiovascular events, recent tracheal radiation therapy, cachexia, poorly controlled hypertension, prolonged QTc interval (corrected QT interval, electrocardiogram), history of significant arrhythmia, untreated brain metastases or recent suicidal ideation” [4] are contraindications to kinase inhibitor therapy.

In the case of PDTCs and ATCs the effective treatment options are limited. Recently a case report of a patient with metastatic PDTC with an *EGFR* mutation was reported [135]. The patient responded to treatment with selective EGFR inhibitor (erlotinib), with PFS longer than 11 months. Although many authors interpret the presence of *EGFR* mutations as associated with primary lung carcinoma with thyroid metastases, Lote and colleagues suggested that these alterations may point primary TC [135]. However, the questions whether specific EGFR inhibitors will be effective in EGFR-mutation positive PDTC cases remains open.

### 3.1. Role of Tumor Status in Prediction of MKI Therapy Success

Since *BRAF* V600E mutation is the most frequent genetic alteration in PTC, inhibitors of this altered kinase has been extensively studied as possible drugs for patients with PTCs harboring this mutation. Promising results were obtained for vemurafenib and dabrafenib, however, like in the case of all kinase inhibitors, treatment resistance is developed [129]. Many potential mechanisms explaining resistance to MKIs therapy have been already proposed. One of the recent studies described the co-occurrence of *BRAF* V600E mutation with either high copy number gain of *MCL1* or loss of *CDKN2A* in metastatic *BRAF*-positive PTC cells associated with primary resistance to vemurafenib [136]. After usage of a combined therapy consisting of vemurafenib and BCL2/MCL1 inhibitor a better response with increased *BRAF* V600E-PTC cells death, comparing to a single agent treatment, was observed. An alternative resistance mechanism to vemurafenib in *BRAF*-positive PTC was described by Danysh et al. [137]. The authors observed acquisition of *KRAS* G12D mutation in a subpopulation of *BRAF*-mutant PTC cells, as a consequence of a long-term vemurafenib pressure. Appearance of this additional alteration led to the loss of sensitivity not only to vemurafenib but also to other kinase inhibitors. KRAS, when activated, uses CRAF as an alternative signal receiver in the RAS/MEK/ERK pathway (MEK- mitogen-activated protein kinase kinase; ERK- extraxellular signal-regulated kinase). It has been demonstrated in melanoma and lung cancer cells that MEK activated by CRAF is less sensitive to MEK inhibitors than, when the activation is via *BRAF* V600E [138]. We cannot exclude that an acquired treatment resistance may influence TC progression to more aggressive types (such as PDTC or ATC). However, this observation needs further studies. In melanoma patients, the usage of BRAF inhibitors may lead to the development of secondary cutaneous squamous cell carcinoma. Forty percent of these patients display *HRAS* mutations [139]. As presented in several studies, BRAF-selective inhibitors are active in *BRAF*-mutant cells but not effective in *BRAF* wild type ones as well as in cells with co-existing *RAS* mutations. Recent study of Chen et al. showed also that these inhibitors are not able to effectively inhibit RAF-MEK-ERK signaling in cells with *RAF* in-frame deletions [140]. The authors found that *BRAF* in frame deletions near the αC-helix region were predicted to lock the helix in the active αC-helix-in conformation that favored dimer formation. These alterations were mutually exclusive with *RAS* mutations and *BRAF* V600E mutation, suggesting their role as potential oncogenes. What is important, cells presenting *RAF* in-frame deletions, although refractory to vemurafenib, were sensitive to the LY3009120 inhibitor. This is due to distinct mechanisms of action of these two inhibitors, with vemurafenib binding to one of two promoters of RAF dimers, and dLY3009120 binding to both promoters of RAF dimers. Chen and colleagues pointed to deletions near the αC-helix domain of protein kinase since, apart from BRAF, these were also detected in EGFR (in non-small cell lung cancer) and HER2 (in breast cancer), which were also targets for anticancer treatment, suggesting these deletions as common mechanism of kinase activation in tumorigenesis.

As mentioned earlier most TKIs, including sorafenib and lenvatinib, are multitargeted drugs, which development resulted from the need to block different signaling pathways to make impossible the escape of cancer cells from therapy. Currently there are no prognostic or predictive biomarkers in MKI treatment of RR-DTCs. However, the *BRAF* V600E mutation is among the candidates. A retrospective study of de la Fouchardiere et al., concerning RR-DTCs, demonstrated that *BRAF*-positive population experienced a significant benefit from TKI treatment, with the median PFS of 34.7 months, comparing to 11.6 months observed in TKI-treated *BRAF* wild-type patients [141]. Surprisingly, *BRAF*-positive RR-DTC patients receiving sorafenib, a non-selective BRAF inhibitor, did not achieve a better treatment outcome than *BRAF*-wild type group. On the other hand, Tahara et al. showed that neither *BRAF* nor *RAS* mutations were prognostic or predictive markers in DTC population of DTC, in contrast to *BRAF* wild type, that may be a poor prognostic factor in patients with RR-PTC [142].

Papers published recently may point a potential impact of molecular background on the effectiveness of MKIs therapy. However, despite of all data given above, the tumor molecular status in TC patients is not taken into consideration, when MKI treatment starts. Further studies are required.

### 3.2. Unsuccessful Attempts of ATC Treatment—Are There Any Chances for Improvement?

ATC, accounting for about 2% of all TCs, represents the most aggressive type. The six-month survival rate is lower than 20% with only 10% of patients surviving at one year [143,144]. According to the TNM classification ATCs are always staged IV and are subdivided into: IVA, intrathyroidal tumors; IVB, extrathyroidal extension but without distant metastases; and IVC, the presence of distant metastases. Most ATCs are detected in stage IVB with unresectable extrathyroidal mass. Despite of many attempts with different drugs there are still no hopes for a better future. The outcomes of chemotherapy with single or multiple agents, radiotherapy, and combined treatment are disappointing and such treatment did not improve survival [145]. We hope new therapeutic strategies based on a molecular ATC background will be promising. It has been demonstrated that 47% of patients diagnosed with ATC had previous or concurrent DTC [146] indicating that a high percent of ATCs develop through dedifferentiation of DTCs. Thus, it is not surprising that genomic alterations observed in DTCs were also present in ATCs and may be used as potential targets. There are some single reports that show a positive response to such treatment in ATC. Rosove et al. reported a dramatic response to vemurafenib in a patient with ATC positive for *BRAF* V600E mutation A complete resolution of the lung metastatic disease was observed [147]. Since approximately 20% to 40% ATCs display this *BRAF* alteration it seems reasonable to evaluate the presence of *BRAF* mutation in ATC cells because patients could benefit from BRAF inhibitors therapy [143]. However, we should to keep in mind that *RAS* mutations are also prevalent in ATCs and, as it was mentioned earlier, their co-existence with *BRAF* V600E mutation led to resistance to vemurafenib. There are no RAS-specific inhibitors, but currently other downstream members, such as mitogen-activated protein kinase, are being studied as potential targets.

The most common alterations in ATC concern *TP53* gene (mutated in 50% to 80% of ATC cases) [143]. However, no effective strategy has been developed to replace the lost of this tumor suppressor functions. Mutations in *TP53* gene can be activated by up-regulated β-catenin, encoded by *CTNNB1* gene, mutated in 5% to 60% of ATCs. β-catenin is a key downstream member of the canonical Wnt signaling pathway, one of the fundamental pathways that directs cell proliferation, cell polarity and cell fate determination during embryonic development and tissue homeostasis, and involved in carcinogenesis [148]. That is why the extensive studies are being conducted to find out the strategies of aberrant Wnt/β-catenin inhibition. Non-steroidal anti-inflammatory drugs, demonstrated the ability to reduce the incidence of different types of cancers and acted via suppression of Wnt pathway by induction of β-catenin degradation or small molecule inhibitors [149]. Among other genes altered frequently in ATC are *PIK3CA* (10% to 20% of ATC cases), *PTEN* (5% to 15%) or *AKT1* (5% to 10%). *PIK3CA* and *AKT1* are of major interest for targeted drug therapy in ATC since the first one is involved in regulation of cell cycle progression, adhesion, and motility and the latter promotes resistance to radiation therapy and standard chemotherapy.

Genomic characterization of ATC is needed in order to find markers that would enable development of new individualized effective therapeutics, increase sensitivity to drugs and overcome resistance. Regardless of encouraging preclinical data three different MKIs administered in ATC patient did not improve the outcomes [150,151,152]. We hope that searching for an effective treatment against ATC is not an impossible dream.

## 4. Medullary Thyroid Carcinoma

MTC, although develops in the same gland, constitutes a distinct neoplastic disease that differs with cancer origin (DTC and ATC arise from follicular cell, whereas MTC from parafollicular C cell), molecular pathogenesis, tumor aggressiveness, treatment modalities and finally long-term outcomes. The progress in MTC diagnostics and treatment is less impressive than in DTC. There are still many unresolved questions.

### 4.1. Genotype-Driven Management of Healthy Individuals and Patients with Cancer in MTC

MTC occurs either sporadically or as an inherited disease. The majority of MTC cases, nearly 75%, are sporadic. Noteworthy, sporadic MTC is very rare in children and most of MTCs diagnosed in this group are hereditary [153].

Familiar MTC is inherited in an autosomal dominant pattern and constitutes a part of multiple endocrine neoplasia type 2 (MEN2) syndrome [154,155]. There are two clinically distinct types of MEN2 syndrome: MEN2A and MEN2B. MEN2A is defined by the presence of MTC, primary hyperparathyroidism (PHP) and pheochromocytoma (PHEO). MTC is diagnosed in nearly 100% of patients, whereas the risk of PHEO and PHP is substantially lower, 50% and 20–30%, respectively. Clinical manifestation of MEN2B syndrome involves MTC, PHEO and typical clinical features with marfanoid habitus and multiple mucosal neuromas among others [154,156].

Hereditary MTC is caused by gain of function mutations in the *RET* proto-oncogene. The type of mutation suggests its clinical course due to strong genotype–phenotype correlation [154,155,157]. In the majority of MEN2A cases, *RET* mutations concern one of five cysteines in its extracellular cysteine-rich domain and they are localized within the exons 10–11. *RET* mutation in the codon 634 is the most characteristic one in MEN2A syndrome. About 95% of MEN2 B cases is related to mutation localized within the intracellular RET tyrosine kinase domain, in codon 918, exon 16 [155,157,158]. Both MEN2A and MEN2B demonstrate a high penetrance, however MEN2B is characterized by a much earlier MTC onset (first years of life) and a worse prognosis [159,160].

Genetic testing for *RET* mutations, widely implemented in a daily practice, significantly reduced the age of MTC diagnosis in affected families and influenced the management [161,162]. The importance of such diagnostics is proved by the fact that an inherited disease was detected in nearly 10% of apparently sporadic MTC patients [163]. According to the current MTC ATA guidelines, *RET* mutations are stratified based on of genotype–phenotype correlations, the penetrance of *RET* mutations and aggressiveness into three MTC risk levels: moderate, high and the highest [162]. This is mainly related to timing of a prophylactic thyroidectomy in *RET* mutation carriers. Children with the highest MTC risk in the course of MEN2B should undergo *RET* genetic testing and surgery during the first year of life. Considering high-risk children (the presence of *RET* mutation in codon 634 of exon 11) the *RET* gene should be tested below the age of five years. If the mutation is found patients have to undergo thyroidectomy. Children with a moderate risk should also undergo genetic testing below the age of five years, however the decision concerning prophylactic thyroidectomy depends on serum Ct level.

*RET* somatic mutations are present in about 60% of sporadic MTC cases [164]. Currently, molecular analyses of *RET* somatic mutations are not routinely performed in sporadic cancer. It is well known that somatic *RET* mutation in the codon 918 is related to poorer outcome [165]. Nevertheless, some clinical trials demonstrated that those patients showed a better response to MKI treatment [166].

### 4.2. The Role of Calcitonin in MTC Monitoring

Calcitonin (Ct) is a sensitive and specific marker used in the diagnostics and monitoring of MTC patients. Only <1% of MTCs are low-differentiated cancers not producing Ct. The basic Ct level may be slightly elevated in patients with renal failure, autoimmune diseases, hyperparathyroidism, FTC, some neuroendocrine pancreatic cancers, prostate cancer or lung cancer [162,167]. However, in cancers different than MTC, calcitonin concentration does not increase in stimulation tests (using calcium or pentagastrin).

Ct level is related to the stage of the disease. It is used to assess MTC advancement and prognosis as well as in the follow-up of a hereditary (associated with the germline mutation of the *RET* proto-oncogene) and sporadic MTC. Both basic and stimulated Ct concentrations influence the timing of prophylactic thyroidectomy in *RET* mutation carriers [162]. This is a significant change compared to the 2009 ATA MTC guidelines where the assessment of calcitonin level was not considered and the time of the prophylactic thyroidectomy depended only on the type of mutation [168]. The European researchers previously indicated the necessity of such approach [169,170].

Postoperative assessment of Ct concentration and carcinoembryonic antigen (CEA) is recommended in all MTC patients. A normalization of serum Ct concentration after surgery indicates an excellent treatment outcome [171,172]. Ten-year overall survival in patients with normal Ct level after total thyroidectomy with an adequate range of cervical lymphadenectomy was nearly 100% [172,173]. However, 80% of patients with palpable MTC and 50% with nonpalpable macroscopic MTC showed persistent elevated serum Ct level after the operation regardless of radical surgical approach. MTC relapse is diagnosed in more than 50% of them during a mean 10-year follow-up [66]. It should be emphasized that not only an absolute Ct value but also its dynamics plays an important role in MTC follow-up. Numerous studies, recently published demonstrated that Ct and CEA doubling time were significant MTC prognostic factors indicating a poor outcome [162,174]. Gawlik et al. reported that Ct and CEA doubling time shorter than 24 months were related to much worse recurrent-free and overall survival [174], whereas according to a meta-analysis, published by Dutch authors, the highest predictive power was found for the doubling time classification 0–1 year vs. > 1 year [175].

If a biochemical remission is not obtained, the source of elevated calcitonin level should be localized. However, imaging studies are useless if Ct level is below 150 pg/mL, because of a very low probability of the detection and localization of MTC recurrence. ATA recommends the assessment of Ct and CEA levels every 3–6 months to determine their doubling time [162]. If an increase in Ct level exceeds 150 pg/mL imaging studies to detect MTC recurrence are recommended [162]. Neck ultrasound should be performed first [176] as nodal recurrence is the most common location of relapse, particularly when only a slight increase in Ct concentration is noticed [177]. The early detection and adequate treatment of MTC relapse has an important impact on disease prognosis and patient survival. The analysis of the results of reoperation due to nodal recurrence revealed that if Ct level before reoperation was > 1000 pg/mL, a final biochemical remission was obtained only in 1 of 76 patients. When Ct level is higher than 500 pg/mL additional imaging studies including chest and abdominal CT/MRI and PET (FDG-PET CT or F-DOPA PET CT) should be performed to confirm dissemination [162]

Based on expert opinions, ATA proposes a “watchful waiting” strategy in patients with sporadic MTC diagnosed after lobectomy if a postoperative normalization of calcitonin level is obtained and such approach is accepted by the patients [162].

### 4.3. A Place for MKI Treatment in Advanced MTC

Multikinase inhibitors have been successfully introduced during recent 10 years into the treatment of advanced MTC, both hereditary and sporadic disease. Similar to in RR-DTC, numerous studies with different MKIs have been already carried out [129] however only two drugs proved a beneficial impact on PFS in phase 3, randomized, placebo-controlled trials: vandetanib (ZETA study) [178] and cabozantinib (EXAM study) [179]. Vandetanib therapy in MTC patients with advanced disease resulted in a significant prolongation of PFS in comparison to placebo, 30.5 months versus 19.3 months, respectively [178]. Similarly, a significantly longer PFS than the in placebo group was obtained in MTC patients, who were given cabozantinib, 11.2 months versus 4.0 months, respectively [179]. ZETA and EXAM trials were carried out in locally advanced or disseminated, hereditary and sporadic MTC. Both studies enrolled a similar number of MTC patients, 331 and 330 respectively. There were two important differences between these trials. The first one concerned inclusion criteria. In ZETA study vandetanib was administered in patients with advanced MTC, regardless whether the disease was progressive or not [178]. While, EXAM study enrolled MTC population with a highly aggressive disease, showing an objective progression within 14 months before randomization [179]. Therefore, the differences in PFS between both studies should not be interpreted as a better vandetanib efficacy. The other disparity was related to study design. Upon progression patients from ZETA trial could receive open-label vandetanib, whereas in EXAM study crossover was not allowed. Both trials failed to demonstrate a beneficial impact of these MKIs on overall survival. Only a subgroup analysis, carried out in EXAM population showed that MTC patients with RET M918T mutation in tumor cells treated with cabozantinib had longer overall survival than placebo group, 44.3 months versus 18.9 months in the placebo group, respectively [166].

MKIs administration does not constitute a curative therapy for MTC patients either. Thus, the necessity of life-long treatment, comorbidities and potential MKI-related complications should be considered in the decision-making process to treat a patient or not. Both the European and ATA MTC guidelines emphasize that such therapy should be limited to patients clinically symptomatic or with a progressive disease [162,180]. The question whether a short Ct doubling time, pointing a poor outcome, constitutes an indication for MKIs administration remains open.

## 5. Conclusions

Regardless of a significant progress in DTC molecular diagnostics, searching for reliable molecular prognostic markers is an important task for the near future.

The current ATA guidelines, which constitute a significant progress in the understanding of evidence-based DTC management, allow to be adapted by a particular center according to its own epidemiological status and legal position. Such possibility is of a greater importance because it results in their much wider use in clinical practice.

ATC is still a challenge for medical care system. We hope that recent advances in molecular understanding of its pathogenesis finally result in a better treatment outcome.

One of the greater advantage of the current ATA MTC guidelines is the necessity to adapt a clinical management not only to the type of *RET* mutation but also to calcitonin concentration. It has to be considered by endocrine surgeons.

## Figures and Tables

**Table 1:** Comparison of two commercially available molecular tests for indeterminate thyroid fine-needle aspiration specimens.

Type of the Test	Afirma	ThyroSeq
	“Rule out”	*“Rule in” and “Rule out”
Methodology	mRNA gene expression (microarray analysis)	next-generation sequencing
Cytology interpretation	Performed in Veracyte laboratory with exception of few academic centers authorized to carry cytology evaluation and submit samples only for molecular testing	Performed by local cytopathologists or centralized laboratory
Sample required	2 dedicated FNA passes	1–2 drops from first pass, if sufficiently cellular
Test report	benign/suspicious	specific mutation/rearrangement
Strength	high NPV; validated in a blinded multicenter prospective trial	high NPV and PPV
Limitations	low PPV; problems with correct classification of Hürthle cell lesions; trained before the reclassification of EFVPTC as benign NIFTP; reclassification of EFVPTC as benign tumors leads to decrease of PPV	limited validation data; increased risk of “false positive” cases associated with the extended mutational profile of the test; the knowledge of the test result before the histopathological assessment in validation study; reclassification of EFVPTC as benign tumors leads to decrease of PPV

* A low pretest risk of malignancy and low PPV makes the tool not satisfactory as “rule in” test.

**Table 2:** The extent of surgery in DTC according to the current ATA guidelines [4].

Extent of Thyroid Resection		Extent of Central Lymph Node Dissection		
Total/Near Total Thyroidectomy	Lobectomy	Therapeutic	Prophylactic (ipsi or Bilateral)	None
Tumor size >4 cm (cT3)	Unifocal, intrathyroidal PMTC without lymph node evidence and distant metastases (cT1aN0M0)	Clinically involved central lymph nodes	Advanced PTC (T3, T4) without involved lymph nodes (cN0)	Low-advanced (T1 or T2), noninvasive, clinically node-negative papillary thyroid carcinoma (cN0)
Gross extrathyroidal extension (cT4)	Optionally: thyroid cancer: >1 cm <4 cm (cT1b, cT2), without lymph nodes (cN0) and distant metastases (M0)		Clinically or biopsy proven metastasis in lateral lymph nodes (N1b)	Most FTCs
Metastatic or suspicious cervical lymph nodes or distant metastases			Information necessary to plan further steps in therapy	
Patient or therapeutic team preference in cT1-T2N0M0 stage				
Older age (>45 years), contralateral thyroid nodules, personal history of radiation therapy to the head and neck, familial DTC history may constitute criteria for a bilateral procedure				

**Table 3:** Summary of the most important molecular markers of thyroid cancers.

Molecular Marker (Signaling Pathway)	Type of Thyroid Cancer	Literature
Indolent thyroid cancers		
Rare *BRAF* alterations (like K601E mutation) (MAPK signaling)	Mainly in FVPTCs and PTMCs	[75,126]
*KRAS* mutations (MAPK signaling)	FVPTCs with favorable clinical and sonographical profile	[127,128]
Aggressive follicular cell derived thyroid cancers		
*BRAF* V600E mutation (MAPK signaling)	PTCs	[76,77,78,79,80,81,82,84,87,88,89,90]
*TERT* promoter mutations (C228T and C250T) (MAPK signaling, WNT signaling)	According to the frequency, starting from the highest: ATC > PDTC > FTC > PTC; In most PTC cases coexisting with *BRAF* V600E mutation	[97,98,99,100,101]
*TP53* inactivating mutations (TP53 signaling)	Mainly in ATCs and PDTCs; also present in a small fraction of aggressive PTCs and FTCs	[52,103]
*CTNNB1*, *APC* and *AXIN1* mutations (WNT signaling)	Mainly in ATC	[104,105,106]
*PTEN*, *AKT1* and *PIK3CA* mutations (PI3K/AKT signaling)	Advanced TCs, mostly in ATCs	[103]
miR-146b	Aggressive BRAF-positive PTCs	[110]
* *RAS* mutations (MAPK signaling)	Present in high fraction of ATCs and PDTCs, but also in FTCs	[76]
Other follicular cell derived thyroid cancers		
*RET/PTC* rearrangements (MAPK pathway)	Molecular hallmark of radiation- induced PTCs	[8,19]

* A controversial role as a molecular marker of aggressive TCs because of their presence in FA and NIFTP.

## Abbreviations

ATA	American Thyroid Association
ATC	Anaplastic thyroid carcinoma
AUS	Atypia of undetermined significance
BRAF	B-Raf proto-oncogene, serine/threonine kinase
CEA	Carcinoembryonic antigen
Ct	Calcitonin
CVPTC	Classical variant of papillary thyroid carcinoma
DTC	Differentiated thyroid carcinoma
EANM	European Association of Nuclear Medicine
EFC	Encapsulated follicular thyroid carcinoma
EFVPTC	Encapsulated follicular variant of papillary thyroid carcinoma
ERK	Extracellular signal-regulated kinase
FA	Follicular adenomas
FLUS	Follicular lesion of undetermined significance
FN	Follicular neoplasm
FNAB	Fine-needle aspiration biopsy
FTC	Follicular thyroid carcinoma
FVPTC	Follicular variant of papillary thyroid carcinoma
GEC	Afirma Gene Expression Classifier
HT	Hashimoto’s thyroiditis
ITN	Incidental thyroid nodule
MAPK	Mitogen-activated protein kinase
MEK	Mitogen-activated protein kinase kinase
MEN2	Multiple endocrine neoplasia type 2
MKIs	Multi kinase inhibitors
MTC	Medullary thyroid carcinoma
NCCN	National Comprehensive Cancer Network
NIFTP	Noninvasive follicular thyroid neoplasm with papillary-like nuclear features
NPV	Negative predictive value
OS	Overall survival
QTc	Corrected QT interval (electrocardiogram)
PBDEs	Polybrominated diphenyl etherflame retardants
PDTC	Poorly differentiated thyroid carcinoma
PFS	Progression-free survival
PHEO	Pheochromocytoma
PHP	Primary hyperparathyroidism
PI3K	Phosphatidylinositol 3-kinase
PPV	Positive predictive value
PTC	Papillary thyroid carcinoma
PTMC	Papillary microcarcinoma
RAI	Radioactive iodine
RAS	Ras proto-oncogene, GTPase
RET	Ret proto-oncogene
RR-DTC	Radioiodine-refractory differentiated thyroid carcinoma
SEER	Surveillance, Epidemiology, and End Results Program
SFN	Suspicious for follicular neoplasm
SUSP	Suspicious for malignancy
TC	Thyroid cancer
TCGA	The Cancer Genome Atlas
TKIs	Tyrosine kinase inhibitors
TKs	Tyrosine kinases
TNM	Tumor, Node, and Metastasis staging
TSVPTC	Papillary thyroid carcinoma with trabecular-solid pattern of growth
US	United States of America

## References

[1] National Cancer Institute Surveilance, Epidemiology, and End Results Program. https://seer.cancer.gov/statfacts/html/thyro.html.

[2] Rahib L., Smith B.D., Aizenberg R., Rosenzweig A.B., Fleshman J.M., Matrisian L.M. (2014). Projecting cancer incidence and deaths to 2030: The unexpected burden of thyroid, liver, and pancreas cancers in the United States. Cancer Res..

[3] Heller K.S. (2007). Do all cancers need to be treated? The role of thyroglobulin in the management of thyroid cancer: The 2006 Hayes Martin lecture. Arch. Otolaryngol. Head Neck Surg..

[4] Haugen B.R., Alexander E.K., Bible K.C., Doherty G.M., Mandel S.J., Nikiforov Y.E., Pacini F., Randolph G.W., Sawka A.M., Schlumberger M. (2016). 2015 American Thyroid Association Management Guidelines for Adult Patients with Thyroid Nodules and Differentiated Thyroid Cancer: The American Thyroid Association Guidelines Task Force on Thyroid Nodules and Differentiated Thyroid Cancer. Thyroid.

[5] Verburg F.A., Aktolun C., Chiti A., Frangos S., Giovanella L., Hoffmann M., Iakovou I., Mihailovic J., Krause B.J., Langsteger W. (2016). Why the European Association of Nuclear Medicine has declined to endorse the 2015 American Thyroid Association management guidelines for adult patients with thyroid nodules and differentiated thyroid cancer. Eur. J. Nucl. Med. Mol. Imaging.

[6] Verburg F.A., Luster M., Giovanella L. (2017). Adjuvant post-operative I-131 therapy in differentiated thyroid carcinoma: Are the 2015 ATA guidelines an exact science or a dark art?. Eur. J. Nucl. Med. Mol. Imaging.

[7] Davies L., Welch H.G. (2014). Current thyroid cancer trends in the United States. JAMA Otolaryngol. Head Neck Surg..

[8] Elisei R. (2014). Molecular profiles of papillary thyroid tumors have been changing in the last decades: How could we explain it?. J. Clin. Endocrinol. Metab..

[9] Vigneri R., Malandrino P., Vigneri P. (2015). The changing epidemiology of thyroid cancer: Why is incidence increasing?. Curr. Opin. Oncol..

[10] Udelsman R., Zhang Y. (2014). The epidemic of thyroid cancer in the United States: The role of endocrinologists and ultrasounds. Thyroid.

[11] Liu Y., Su L., Xiao H. (2017). Review of Factors Related to the Thyroid Cancer Epidemic. Int. J. Endocrinol..

[12] Corso C., Gomez X., Sanabria A., Vega V., Dominguez L.C., Osorio C. (2014). Total thyroidectomy versus hemithyroidectomy for patients with follicular neoplasm. A cost-utility analysis. Int. J. Surg..

[13] Hoang J.K., Langer J.E., Middleton W.D., Wu C.C., Hammers L.W., Cronan J.J., Tessler F.N., Grant E.G., Berland L.L. (2015). Managing incidental thyroid nodules detected on imaging: White paper of the ACR Incidental Thyroid Findings Committee. J. Am. Coll. Radiol..

[14] Harach H.R., Franssila K.O., Wasenius V.M. (1985). Occult papillary carcinoma of the thyroid. A “normal”; finding in Finland. A systematic autopsy study. Cancer.

[15] Wang T.S., Goffredo P., Sosa J.A., Roman S.A. (2014). Papillary thyroid microcarcinoma: An over-treated malignancy?. World J. Surg..

[16] Nikiforov Y.E., Seethala R.R., Tallini G., Baloch Z.W., Basolo F., Thompson L.D.R., Barletta J.A., Wenig B.M., Al Ghuzlan A., Kakudo K. (2016). Nomenclature Revision for Encapsulated Follicular Variant of Papillary Thyroid Carcinoma: A Paradigm Shift to Reduce Overtreatment of Indolent Tumors. JAMA Oncol..

[17] Chen A.Y., Jemal A., Ward E.M. (2009). Increasing incidence of differentiated thyroid cancer in the United States, 1988–2005. Cancer.

[18] Vergamini L.B., Frazier A.L., Abrantes F.L., Ribeiro K.B., Rodriguez-Galindo C. (2014). Increase in the incidence of differentiated thyroid carcinoma in children, adolescents, and young adults: A population-based study. J. Pediatr..

[19] Jung C.K., Little M.P., Lubin J.H., Brenner A.V., Wells S.A.J., Sigurdson A.J., Nikiforov Y.E. (2014). The increase in thyroid cancer incidence during the last four decades is accompanied by a high frequency of BRAF mutations and a sharp increase in RAS mutations. J. Clin. Endocrinol. Metab..

[20] Smyth P., Finn S., Cahill S., O’Regan E., Flavin R., O’Leary J.J., Sheils O. (2005). ret/PTC and BRAF act as distinct molecular, time-dependant triggers in a sporadic Irish cohort of papillary thyroid carcinoma. Int. J. Surg. Pathol..

[21] Mathur A., Moses W., Rahbari R., Khanafshar E., Duh Q.-Y., Clark O., Kebebew E. (2011). Higher rate of BRAF mutation in papillary thyroid cancer over time: A single-institution study. Cancer.

[22] Romei C., Fugazzola L., Puxeddu E., Frasca F., Viola D., Muzza M., Moretti S., Nicolosi M.L., Giani C., Cirello V. (2012). Modifications in the papillary thyroid cancer gene profile over the last 15 years. J. Clin. Endocrinol. Metab..

[23] Guan H., Ji M., Bao R., Yu H., Wang Y., Hou P., Zhang Y., Shan Z., Teng W., Xing M. (2009). Association of high iodine intake with the T1799A BRAF mutation in papillary thyroid cancer. J. Clin. Endocrinol. Metab..

[24] Weihrauch M., Benick M., Lehner G., Wittekind M., Bader M., Wrbitzk R., Tannapfel A. (2001). High prevalence of K-ras-2 mutations in hepatocellular carcinomas in workers exposed to vinyl chloride. Int. Arch. Occup. Environ. Health.

[25] Porta M., Crous-Bou M., Wark P.A., Vineis P., Real F.X., Malats N., Kampman E. (2009). Cigarette smoking and K-ras mutations in pancreas, lung and colorectal adenocarcinomas: Etiopathogenic similarities, differences and paradoxes. Mutat. Res..

[26] Zhang Y., Guo G.L., Han X., Zhu C., Kilfoy B.A., Zhu Y., Boyle P., Zheng T. (2008). Do Polybrominated Diphenyl Ethers (PBDEs) Increase the Risk of Thyroid Cancer?. Biosci. Hypotheses.

[27] Leitzmann M.F., Brenner A., Moore S.C., Koebnick C., Park Y., Hollenbeck A., Schatzkin A., Ron E. (2010). Prospective study of body mass index, physical activity and thyroid cancer. Int. J. Cancer.

[28] Rinaldi S., Lise M., Clavel-Chapelon F., Boutron-Ruault M.-C., Guillas G., Overvad K., Tjonneland A., Halkjaer J., Lukanova A., Kaaks R. (2012). Body size and risk of differentiated thyroid carcinomas: Findings from the EPIC study. Int. J. Cancer.

[29] Larson S.D., Jackson L.N., Riall T.S., Uchida T., Thomas R.P., Qiu S., Evers B.M. (2007). Increased incidence of well-differentiated thyroid cancer associated with Hashimoto thyroiditis and the role of the PI3k/Akt pathway. J. Am. Coll. Surg..

[30] Rajoria S., Suriano R., Shanmugam A., Wilson Y.L., Schantz S.P., Geliebter J., Tiwari R.K. (2010). Metastatic phenotype is regulated by estrogen in thyroid cells. Thyroid.

[31] Derwahl M., Nicula D. (2014). Estrogen and its role in thyroid cancer. Endocr. Relat. Cancer.

[32] Zahid M., Goldner W., Beseler C.L., Rogan E.G., Cavalieri E.L. (2013). Unbalanced estrogen metabolism in thyroid cancer. Int. J. Cancer.

[33] Luo J., Hendryx M., Manson J.E., Liang X., Margolis K.L. (2016). Hysterectomy, Oophorectomy, and Risk of Thyroid Cancer. J. Clin. Endocrinol. Metab..

[34] Wartofsky L. (2016). A rose by any name surely does smell just as sweetly: The controversy over revised nomenclature for encapsulated follicular variant papillary carcinoma. J. Transl. Intern. Med..

[35] Thompson L.D. (2016). Ninety-four cases of encapsulated follicular variant of papillary thyroid carcinoma: A name change to Noninvasive Follicular Thyroid Neoplasm with Papillary-like Nuclear Features would help prevent overtreatment. Mod. Pathol..

[36] Seethala R.R., Asa S.L., Bullock M.J., Carty S.E., Hodak S.P., McHugh J.B., Nikiforov Y.E., Pettus J., Richardson M.S., Shah J. (1999). Protocol for the Examination of Specimens From Patients With Carcinomas of the Thyroid Gland. Arch. Pathol. Lab. Med..

[37] Johnson S.J., Stephenson T.J. NIFTP Addendum to the RCPath Dataset for Thyroid Cancer Histopathology Reports. http://www.niftp.org/NIFTP.pdf.

[38] Xu B., Tallini G., Ghossein R.A. (2017). Noninvasive Follicular Thyroid Neoplasm with Papillary-Like Nuclear Features: Historical Context, Diagnosis, and Future Challenges. Endocr. Pathol..

[39] Farat N., Xu B., Onernek A., Tuttle R., Roman B., Katabi N., Nose V., Sadow P., Tallini G., Faqiun W. Should subcentimeter non-invasive encapsulated, follicular variant of papillary thyroid carcinoma (NI-EFV PTC) be included as noninvasive follicular thyroid neoplasms with papillary-like nuclear features (NIFTP)?. Proceedings of the USCAP 106th Annual Meeting.

[40] Xu B., Tallini G., Scognamiglio T., Roman B.R., Tuttle R.M., Ghossein R.A. (2017). Outcome of Large Noninvasive Follicular Thyroid Neoplasm with Papillary-Like Nuclear Features. Thyroid.

[41] Poller D.N., Nikiforov Y.E. (2016). Non-invasive follicular thyroid neoplasm with papillary-like nuclei: Reducing overtreatment by reclassifying an indolent variant of papillary thyroid cancer. J. Clin. Pathol..

[42] Ganly I., Wang L., Tuttle R.M., Katabi N., Ceballos G.A., Harach H.R., Ghossein R. (2015). Invasion rather than nuclear features correlates with outcome in encapsulated follicular tumors: Further evidence for the reclassification of the encapsulated papillary thyroid carcinoma follicular variant. Hum. Pathol..

[43] Lastra R.R., Pramick M.R., Crammer C.J., LiVolsi V.A., Baloch Z.W. (2014). Implications of a suspicious afirma test result in thyroid fine-needle aspiration cytology: An institutional experience. Cancer Cytopathol..

[44] Wang C.-C.C., Friedman L., Kennedy G.C., Wang H., Kebebew E., Steward D.L., Zeiger M.A., Westra W.H., Wang Y., Khanafshar E. (2011). A large multicenter correlation study of thyroid nodule cytopathology and histopathology. Thyroid.

[45] Xing M., Haugen B.R., Schlumberger M. (2013). Progress in molecular-based management of differentiated thyroid cancer. Lancet.

[46] Chudova D., Wilde J.I., Wang E.T., Wang H., Rabbee N., Egidio C.M., Reynolds J., Tom E., Pagan M., Rigl C.T. (2010). Molecular classification of thyroid nodules using high-dimensionality genomic data. J. Clin. Endocrinol. Metab..

[47] Alexander E.K., Kennedy G.C., Baloch Z.W., Cibas E.S., Chudova D., Diggans J., Friedman L., Kloos R.T., LiVolsi V.A., Mandel S.J. (2012). Preoperative diagnosis of benign thyroid nodules with indeterminate cytology. N. Engl. J. Med..

[48] Alexander E.K., Schorr M., Klopper J., Kim C., Sipos J., Nabhan F., Parker C., Steward D.L., Mandel S.J., Haugen B.R. (2014). Multicenter clinical experience with the Afirma gene expression classifier. J. Clin. Endocrinol. Metab..

[49] Nishino M. (2016). Molecular cytopathology for thyroid nodules: A review of methodology and test performance. Cancer Cytopathol..

[50] Zhang M., Lin O. (2016). Molecular Testing of Thyroid Nodules: A Review of Current Available Tests for Fine-Needle Aspiration Specimens. Arch. Pathol. Lab. Med..

[51] Brauner E., Holmes B.J., Krane J.F., Nishino M., Zurakowski D., Hennessey J.V., Faquin W.C., Parangi S. (2015). Performance of the Afirma Gene Expression Classifier in Hurthle Cell Thyroid Nodules Differs from Other Indeterminate Thyroid Nodules. Thyroid.

[52] Nikiforova M.N., Wald A.I., Roy S., Durso M.B., Nikiforov Y.E. (2013). Targeted Next-Generation Sequencing Panel (ThyroSeq) for Detection of Mutations in Thyroid Cancer. J. Clin. Endocrinol. Metab..

[53] Nikiforov Y.E., Carty S.E., Chiosea S.I., Coyne C., Duvvuri U., Ferris R.L., Gooding W.E., Hodak S.P., LeBeau S.O., Ohori N.P. (2014). Highly accurate diagnosis of cancer in thyroid nodules with follicular neoplasm/suspicious for a follicular neoplasm cytology by ThyroSeq v2 next-generation sequencing assay. Cancer.

[54] Nixon I.J. (2016). Wysoko zróżnicowany rak tarczycy—Czy nie leczysz swoich pacjentów zbyt intensywnie?. Endokrynol. Pol..

[55] Allan E., Owens S.E., Waller M.L. (1999). Differentiated thyroid cancer: Lobectomy and radioiodine, a treatment suitable for all cases?. Nucl. Med. Commun..

[56] Bramley M.D., Harrison B.J. (1996). Papillary microcarcinoma of the thyroid gland. Br. J. Surg..

[57] Cohn K.H., Backdahl M., Forsslund G., Auer G., Zetterberg A., Lundell G., Granberg P.O., Lowhagen T., Willems J.S., Cady B. (1984). Biologic considerations and operative strategy in papillary thyroid carcinoma: Arguments against the routine performance of total thyroidectomy. Surgery.

[58] De Montes F.B.G., Huerta H.D., Ortiz M.G., Camacho G.V., Solano F.R. (1999). Comparison of three prognostic indexes in differentiated thyroid cancer. Eur. J. Surg. Oncol..

[59] DeGroot L.J., Kaplan E.L., McCormick M., Straus F.H. (1990). Natural history, treatment, and course of papillary thyroid carcinoma. J. Clin. Endocrinol. Metab..

[60] Mazzaferri E.L. (1999). An overview of the management of papillary and follicular thyroid carcinoma. Thyroid.

[61] Czarniecka A., Jarzab M., Krajewska J., Sacher A., Poltorak S., Wloch J. (2006). The impact of the extent and time of operation on the survival in patients with differentiated thyroid carcinoma (DTC). Endokrynol. Pol..

[62] Cooper D.S., Doherty G.M., Haugen B.R., Hauger B.R., Kloos R.T., Lee S.L., Mandel S.J., Mazzaferri E.L., McIver B., Pacini F. (2009). Revised American Thyroid Association management guidelines for patients with thyroid nodules and differentiated thyroid cancer. Thyroid.

[63] Adam M.A., Goffredo P., Youngwirth L., Scheri R.P., Roman S.A., Sosa J.A. (2016). Same thyroid cancer, different national practice guidelines: When discordant American Thyroid Association and National Comprehensive Cancer Network surgery recommendations are associated with compromised patient outcome. Surgery.

[64] Adam M.A., Pura J., Gu L., Dinan M.A., Tyler D.S., Reed S.D., Scheri R., Roman S.A., Sosa J.A. (2014). Extent of surgery for papillary thyroid cancer is not associated with survival: An analysis of 61,775 patients. Ann. Surg..

[65] Czarniecka A., Poltorak S., Sacher A., Maciejewski A., Krajewska J., Jarzab M., Stobiecka E., Zembala-Nozynska E., Chmielik E., Rusinek D. (2014). Surgical strategy in low advanced differentiated thyroid cancer staged T1N0M0-results of a pilot feasibility study. 38th Annual Meeting of the European Thyroid Association, Santiago de Compostela, Spain, September 6–10, 2014; OP93. Eur. Thyroid J..

[66] Tuttle R.M., Haddad R.I., Ball D.W., Byrd D., Dickson P., Duh Q.-Y., Ehya H., Haymart M., Hoh C., Hunt J.P. (2014). Thyroid carcinoma, version 2.2014. J. Natl. Compr. Cancer Netw..

[67] Perros P., Boelaert K., Colley S., Evans C., Evans R.M., Gerrard Ba G., Gilbert J., Harrison B., Johnson S.J., Giles T.E. (2014). Guidelines for the management of thyroid cancer. Clin. Endocrinol..

[68] Barney B.M., Hitchcock Y.J., Sharma P., Shrieve D.C., Tward J.D. (2011). Overall and cause-specific survival for patients undergoing lobectomy, near-total, or total thyroidectomy for differentiated thyroid cancer. Head Neck.

[69] Mendelsohn A.H., Elashoff D.A., Abemayor E., St John M.A. (2010). Surgery for papillary thyroid carcinoma: Is lobectomy enough?. Arch. Otolaryngol. Head Neck Surg..

[70] Bilimoria K.Y., Bentrem D.J., Ko C.Y., Stewart A.K., Winchester D.P., Talamonti M.S., Sturgeon C. (2007). Extent of surgery affects survival for papillary thyroid cancer. Ann. Surg..

[71] Ito Y., Miyauchi A., Oda H. (2017). Low-risk papillary microcarcinoma of the thyroid: A review of active surveillance trials. Eur. J. Surg. Oncol..

[72] Schlumberger M., Pacini F., Tuttle R. (2016). Initial treatment. Thyroid Tumors.

[73] Schmidbauer B., Menhart K., Hellwig D., Grosse J. (2017). Differentiated Thyroid Cancer-Treatment: State of the Art. Int. J. Mol. Sci..

[74] Jarząb B., Dedecjus M., Handkiewicz-Junak D., Lange D., Lewiński A., Nasierowska-Guttmejer A., Ruchała M., Słowińska-Klencka D., Nauman J., Adamczewski Z. (2016). Diagnostics and Treatment of Thyroid Carcinoma. Endokrynol. Pol..

[75] Agrawal N., Akbani R., Aksoy B.A., Ally A., Arachchi H., Asa S.L., Auman J.T., Balasundaram M., Balu S., Baylin S.B. (2014). Integrated Genomic Characterization of Papillary Thyroid Carcinoma. Cell.

[76] Tavares C., Melo M., Cameselle-Teijeiro J.M., Soares P., Sobrinho-Simoes M. (2016). Endocrine Tumours: Genetic predictors of thyroid cancer outcome. Eur. J. Endocrinol..

[77] Kebebew E., Weng J., Bauer J., Ranvier G., Clark O.H., Duh Q.-Y., Shibru D., Bastian B., Griffin A. (2007). The prevalence and prognostic value of BRAF mutation in thyroid cancer. Ann. Surg..

[78] Kim T.Y., Kim W.B., Rhee Y.S., Song J.Y., Kim J.M., Gong G., Lee S., Kim S.Y., Kim S.C., Hong S.J. (2006). The BRAF mutation is useful for prediction of clinical recurrence in low-risk patients with conventional papillary thyroid carcinoma. Clin. Endocrinol..

[79] Namba H., Nakashima M., Hayashi T., Hayashida N., Maeda S., Rogounovitch T.I., Ohtsuru A., Saenko V.A., Kanematsu T., Yamashita S. (2003). Clinical implication of hot spot BRAF mutation, V599E, in papillary thyroid cancers. J. Clin. Endocrinol. Metab..

[80] Oler G., Cerutti J.M. (2009). High prevalence of BRAF mutation in a Brazilian cohort of patients with sporadic papillary thyroid carcinomas: Correlation with more aggressive phenotype and decreased expression of iodide-metabolizing genes. Cancer.

[81] Elisei R., Ugolini C., Viola D., Lupi C., Biagini A., Giannini R., Romei C., Miccoli P., Pinchera A., Basolo F. (2008). BRAF(V600E) mutation and outcome of patients with papillary thyroid carcinoma: A 15-year median follow-up study. J. Clin. Endocrinol. Metab..

[82] Xing M., Westra W.H., Tufano R.P., Cohen Y., Rosenbaum E., Rhoden K.J., Carson K.A., Vasko V., Larin A., Tallini G. (2005). BRAF mutation predicts a poorer clinical prognosis for papillary thyroid cancer. J. Clin. Endocrinol. Metab..

[83] Fugazzola L., Puxeddu E., Avenia N., Romei C., Cirello V., Cavaliere A., Faviana P., Mannavola D., Moretti S., Rossi S. (2006). Correlation between B-RAFV600E mutation and clinico-pathologic parameters in papillary thyroid carcinoma: Data from a multicentric Italian study and review of the literature. Endocr. Relat. Cancer.

[84] Riesco-Eizaguirre G., Gutierrez-Martinez P., Garcia-Cabezas M.A., Nistal M., Santisteban P. (2006). The oncogene BRAF V600E is associated with a high risk of recurrence and less differentiated papillary thyroid carcinoma due to the impairment of Na^+^/I^−^ targeting to the membrane. Endocr. Relat. Cancer.

[85] Hardee S., Prasad M.L., Hui P., Dinauer C.A., Morotti R.A. (2017). Pathologic Characteristics, Natural History, and Prognostic Implications of BRAFV600E Mutation in Pediatric Papillary Thyroid Carcinoma. Pediatr. Dev. Pathol..

[86] Geng J., Wang H., Liu Y., Tai J., Jin Y., Zhang J., He L., Fu L., Qin H., Song Y. (2017). Correlation between BRAF V600E mutation and clinicopathological features in pediatric papillary thyroid carcinoma. Sci. China Life Sci..

[87] Xing M., Alzahrani A.S., Carson K.A., Viola D., Elisei R., Bendlova B., Yip L., Mian C., Vianello F., Tuttle R.M. (2013). Association between BRAF V600E mutation and mortality in patients with papillary thyroid cancer. JAMA.

[88] Xing M. (2013). Molecular pathogenesis and mechanisms of thyroid cancer. Nat. Rev. Cancer.

[89] Xing M., Alzahrani A.S., Carson K.A., Shong Y.K., Kim T.Y., Viola D., Elisei R., Bendlová B., Yip L., Mian C. (2015). Association Between *BRAF* V600E Mutation and Recurrence of Papillary Thyroid Cancer. J. Clin. Oncol..

[90] Xing M. (2015). *BRAF* Mutation and Thyroid Cancer Recurrence. J. Clin. Oncol..

[91] Abrosimov A., Saenko V., Rogounovitch T., Namba H., Lushnikov E., Mitsutake N., Yamashita S. (2007). Different structural components of conventional papillary thyroid carcinoma display mostly identical BRAF status. Int. J. Cancer.

[92] Ghossein R.A., Katabi N., Fagin J.A. (2013). Immunohistochemical detection of mutated BRAF V600E supports the clonal origin of BRAF-induced thyroid cancers along the spectrum of disease progression. J. Clin. Endocrinol. Metab..

[93] Guerra A., Sapio M.R., Marotta V., Campanile E., Rossi S., Forno I., Fugazzola L., Budillon A., Moccia T., Fenzi G. (2012). The primary occurrence of BRAF(V600E) is a rare clonal event in papillary thyroid carcinoma. J. Clin. Endocrinol. Metab..

[94] Gandolfi G., Sancisi V., Torricelli F., Ragazzi M., Frasoldati A., Piana S., Ciarrocchi A. (2013). Allele percentage of the BRAF V600E mutation in papillary thyroid carcinomas and corresponding lymph node metastases: No evidence for a role in tumor progression. J. Clin. Endocrinol. Metab..

[95] De Biase D., Cesari V., Visani M., Casadei G.P., Cremonini N., Gandolfi G., Sancisi V., Ragazzi M., Pession A., Ciarrocchi A. (2014). High-sensitivity BRAF mutation analysis: BRAF V600E is acquired early during tumor development but is heterogeneously distributed in a subset of papillary thyroid carcinomas. J. Clin. Endocrinol. Metab..

[96] Huang F.W., Hodis E., Xu M.J., Kryukov G.V., Chin L., Garraway L.A. (2013). Highly recurrent TERT promoter mutations in human melanoma. Science.

[97] Liu X., Bishop J., Shan Y., Pai S., Liu D., Murugan A.K., Sun H., El-Naggar A.K., Xing M. (2013). Highly prevalent TERT promoter mutations in aggressive thyroid cancers. Endocr. Relat. Cancer.

[98] Melo M., da Rocha A.G., Vinagre J., Batista R., Peixoto J., Tavares C., Celestino R., Almeida A., Salgado C., Eloy C. (2014). TERT promoter mutations are a major indicator of poor outcome in differentiated thyroid carcinomas. J. Clin. Endocrinol. Metab..

[99] Xing M., Liu R., Liu X., Murugan A.K., Zhu G., Zeiger M.A., Pai S., Bishop J. (2014). BRAF V600E and TERT promoter mutations cooperatively identify the most aggressive papillary thyroid cancer with highest recurrence. J. Clin. Oncol..

[100] Liu X., Qu S., Liu R., Sheng C., Shi X., Zhu G., Murugan A.K., Guan H., Yu H., Wang Y. (2014). TERT promoter mutations and their association with braf v600e mutation and aggressive clinicopathological characteristics of thyroid cancer. J. Clin. Endocrinol. Metab..

[101] Song Y.S., Lim J.A., Choi H., Won J.-K., Moon J.H., Cho S.W., Lee K.E., Park Y.J., Yi K.H., Park D.J. (2016). Prognostic effects of TERT promoter mutations are enhanced by coexistence with BRAF or RAS mutations and strengthen the risk prediction by the ATA or TNM staging system in differentiated thyroid cancer patients. Cancer.

[102] Vinagre J., Almeida A., Populo H., Batista R., Lyra J., Pinto V., Coelho R., Celestino R., Prazeres H., Lima L. (2013). Frequency of TERT promoter mutations in human cancers. Nat. Commun..

[103] Xu B., Ghossein R. (2016). Genomic landscape of poorly differentiated and anaplastic thyroid Carcinoma. Endocr. Pathol..

[104] Garcia-Rostan G., Camp R.L., Herrero A., Carcangiu M.L., Rimm D.L., Tallini G. (2001). Beta-catenin dysregulation in thyroid neoplasms: Down-regulation, aberrant nuclear expression, and CTNNB1 exon 3 mutations are markers for aggressive tumor phenotypes and poor prognosis. Am. J. Pathol..

[105] Garcia-Rostan G., Tallini G., Herrero A., D’Aquila T.G., Carcangiu M.L., Rimm D.L. (1999). Frequent mutation and nuclear localization of beta-catenin in anaplastic thyroid carcinoma. Cancer Res..

[106] Kurihara T., Ikeda S., Ishizaki Y., Fujimori M., Tokumoto N., Hirata Y., Ozaki S., Okajima M., Sugino K., Asahara T. (2004). Immunohistochemical and sequencing analyses of the Wnt signaling components in Japanese anaplastic thyroid cancers. Thyroid.

[107] Landa I., Ibrahimpasic T., Boucai L., Sinha R., Knauf J.A., Shah R.H., Dogan S., Ricarte-Filho J.C., Krishnamoorthy G.P., Xu B. (2016). Genomic and transcriptomic hallmarks of poorly differentiated and anaplastic thyroid cancers. J. Clin. Investig..

[108] Kunstman J.W., Juhlin C.C., Goh G., Brown T.C., Stenman A., Healy J.M., Rubinstein J.C., Choi M., Kiss N., Nelson-Williams C. (2015). Characterization of the mutational landscape of anaplastic thyroid cancer via whole-exome sequencing. Hum. Mol. Genet..

[109] Ringel M.D. (2009). Molecular markers of aggressiveness of thyroid cancer. Curr. Opin. Endocrinol. Diabetes Obes..

[110] Chou C.-K., Liu R.-T., Kang H.-Y. (2017). MicroRNA-146b: A novel biomarker and therapeutic target for human papillary thyroid cancer. Int. J. Mol. Sci..

[111] Guo Z., Hardin H., Montemayor-Garcia C., Asioli S., Righi A., Maletta F., Sapino A., Lloyd R.V. (2015). In situ hybridization analysis of mir-146b-5p and mir-21 in thyroid nodules: Diagnostic implications. Endocr. Pathol..

[112] Yamamoto Y., Maeda T., Izumi K., Otsuka H. (1990). Occult papillary carcinoma of the thyroid. A study of 408 autopsy cases. Cancer.

[113] Davison B.J., Oliffe J.L., Pickles T., Mroz L. (2009). Factors influencing men undertaking active surveillance for the management of low-risk prostate cancer. Oncol. Nurs. Forum.

[114] Gorin M.A., Eldefrawy A., Ekwenna O., Soloway M.S. (2012). Active surveillance for low-risk prostate cancer: Knowledge, acceptance and practice among urologists. Prostate Cancer Prostatic Dis..

[115] Ito Y., Uruno T., Nakano K., Takamura Y., Miya A., Kobayashi K., Yokozawa T., Matsuzuka F., Kuma S., Kuma K. (2003). An observation trial without surgical treatment in patients with papillary microcarcinoma of the thyroid. Thyroid.

[116] Sugitani I., Toda K., Yamada K., Yamamoto N., Ikenaga M., Fujimoto Y. (2010). Three distinctly different kinds of papillary thyroid microcarcinoma should be recognized: Our treatment strategies and outcomes. World J. Surg..

[117] Ito Y., Miyauchi A., Inoue H., Fukushima M., Kihara M., Higashiyama T., Tomoda C., Takamura Y., Kobayashi K., Miya A. (2010). An observational trial for papillary thyroid microcarcinoma in Japanese patients. World J. Surg..

[118] Ito Y., Miyauchi A., Kihara M., Higashiyama T., Kobayashi K., Miya A. (2014). Patient age is significantly related to the progression of papillary microcarcinoma of the thyroid under observation. Thyroid.

[119] Oda H., Miyauchi A., Ito Y., Yoshioka K., Nakayama A., Sasai H., Masuoka H., Yabuta T., Fukushima M., Higashiyama T. (2016). Incidences of unfavorable events in the management of low-risk papillary Microcarcinoma of the Thyroid by Active Surveillance Versus Immediate Surgery. Thyroid.

[120] Fukuoka O., Sugitani I., Ebina A., Toda K., Kawabata K., Yamada K. (2016). Natural history of asymptomatic papillary thyroid microcarcinoma: Time-dependent changes in calcification and vascularity during active surveillance. World J. Surg..

[121] Hay I.D. (2007). Management of patients with low-risk papillary thyroid carcinoma. Endocr. Pract..

[122] Mazzaferri E.L. (2007). Management of low-risk differentiated thyroid cancer. Endocr. Pract..

[123] Bradley N.L., Wiseman S.M. (2017). Papillary thyroid microcarcinoma: The significance of high risk features. BMC Cancer.

[124] Brito J.P., Ito Y., Miyauchi A., Tuttle R.M. (2016). A clinical framework to facilitate risk stratification when considering an active surveillance alternative to immediate biopsy and surgery in papillary microcarcinoma. Thyroid.

[125] Leboulleux S., Tuttle R.M., Pacini F., Schlumberger M. (2016). Papillary thyroid microcarcinoma: Time to shift from surgery to active surveillance?. Lancet Diabetes Endocrinol..

[126] Torregrossa L., Viola D., Sensi E., Giordano M., Piaggi P., Romei C., Materazzi G., Miccoli P., Elisei R., Basolo F. (2016). Papillary thyroid carcinoma with rare exon 15 braf mutation has indolent behavior: A single-institution experience. J. Clin. Endocrinol. Metab..

[127] Kakarmath S., Heller H.T., Alexander C.A., Cibas E.S., Krane J.F., Barletta J.A., Lindeman N.I., Frates M.C., Benson C.B., Gawande A.A. (2016). Clinical, sonographic, and pathological characteristics of ras-positive versus braf-positive thyroid carcinoma. J. Clin. Endocrinol. Metab..

[128] Medici M., Kwong N., Angell T.E., Marqusee E., Kim M.I., Frates M.C., Benson C.B., Cibas E.S., Barletta J.A., Krane J.F. (2015). The variable phenotype and low-risk nature of RAS-positive thyroid nodules. BMC Med..

[129] Krajewska J., Gawlik T., Jarzab B. (2017). Advances in small molecule therapy for treating metastatic thyroid cancer. Expert Opin. Pharmacother..

[130] Brose M.S., Nutting C.M., Jarzab B., Elisei R., Siena S., Bastholt L., de la Fouchardiere C., Pacini F., Paschke R., Shong Y.K. (2014). Sorafenib in radioactive iodine-refractory, locally advanced or metastatic differentiated thyroid cancer: A randomised, double-blind, phase 3 trial. Lancet.

[131] Schlumberger M., Tahara M., Wirth L.J., Robinson B., Brose M.S., Elisei R., Habra M.A., Newbold K., Shah M.H., Hoff A.O. (2015). Lenvatinib versus Placebo in Radioiodine-Refractory Thyroid Cancer. N. Engl. J. Med..

[132] Brose M., Jarzab B., Elisei R., Siena S., Bastholt L., de la Fouchardiere C., Pacini F., Paschke R., Nutting C., Shong Y. (2014). Updated overall survival analysis of patients with locally advanced or metastatic radioiodine-refractory differentiated thyroid cancer treated with sorafenib on phase 3 DECISION trial (ABSTRACT). J. Clin. Oncol..

[133] Brose M.S., Worden F.P., Newbold K.L., Guo M., Hurria A. (2017). Effect of age on the efficacy and safety of lenvatinib in radioiodine-refractory differentiated thyroid cancer in the phase III select trial. J. Clin. Oncol..

[134] Krajewska J., Paliczka-Cieslik E., Jarzab B. (2017). Managing tyrosine kinase inhibitors side effects in thyroid cancer. Expert Rev. Endocrinol. Metab..

[135] Lote H., Bhosle J., Thway K., Newbold K., O’Brien M. (2014). Epidermal growth factor mutation as a diagnostic and therapeutic target in metastatic poorly differentiated thyroid carcinoma: A case report and review of the literature. Case Rep. Oncol..

[136] Duquette M., Sadow P.M., Husain A., Sims J.N., Antonello Z.A., Fischer A.H., Song C., Castellanos-Rizaldos E., Makrigiorgos G.M., Kurebayashi J. (2015). Metastasis-associated MCL1 and P16 copy number alterations dictate resistance to vemurafenib in a BRAFV600E patient-derived papillary thyroid carcinoma preclinical model. Oncotarget.

[137] Danysh B.P., Rieger E.Y., Sinha D.K., Evers C.V., Cote G.J., Cabanillas M.E., Hofmann M.-C. (2016). Long-term vemurafenib treatment drives inhibitor resistance through a spontaneous KRAS G12D mutation in a BRAF V600E papillary thyroid carcinoma model. Oncotarget.

[138] Lito P., Saborowski A., Yue J., Solomon M., Joseph E., Gadal S., Saborowski M., Kastenhuber E., Fellmann C., Ohara K. (2014). Disruption of CRAF-mediated MEK activation is required for effective MEK inhibition in KRAS mutant tumors. Cancer Cell.

[139] Lacouture M.E., Duvic M., Hauschild A., Prieto V.G., Robert C., Schadendorf D., Kim C.C., McCormack C.J., Myskowski P.L., Spleiss O. (2013). Analysis of dermatologic events in vemurafenib-treated patients with melanoma. Oncologist.

[140] Chen S.-H., Zhang Y., Van Horn R.D., Yin T., Buchanan S., Yadav V., Mochalkin I., Wong S.S., Yue Y.G., Huber L. (2016). Oncogenic BRAF Deletions That Function as Homodimers and Are Sensitive to Inhibition by RAF Dimer Inhibitor LY3009120. Cancer Discov..

[141] De la Fouchardiere C., Oussaid N., Derbel O., Decaussin-Petrucci M., Fondrevelle M.-E., Wang Q., Bringuier P.-P., Bournaud-Salinas C., Peix J.-L., Lifante J.-C. (2016). Does Molecular Genotype Provide Useful Information in the Management of Radioiodine Refractory Thyroid Cancers? Results of a Retrospective Study. Target Oncol..

[142] Tahara M., Schlumberger M., Elisei R., Habra M.A., Kiyota N., Paschke R., Dutcus C.E., Hihara T., McGrath S., Matijevic M. (2017). Exploratory analysis of biomarkers associated with clinical outcomes from the study of lenvatinib in differentiated cancer of the thyroid. Eur. J. Cancer.

[143] Pinto N., Black M., Patel K., Yoo J., Mymryk J.S., Barrett J.W., Nichols A.C. (2014). Genomically driven precision medicine to improve outcomes in anaplastic thyroid cancer. J. Oncol..

[144] Schlumberger M., Baudin E., Travagli J.P. (1998). Papillary and follicular cancers of the thyroid. Presse Med..

[145] Smallridge R.C., Ain K.B., Asa S.L., Bible K.C., Brierley J.D., Burman K.D., Kebebew E., Lee N.Y., Nikiforov Y.E., Rosenthal M.S. (2012). American Thyroid Association guidelines for management of patients with anaplastic thyroid cancer. Thyroid.

[146] Demeter J.G., De Jong S.A., Lawrence A.M., Paloyan E. (1991). Anaplastic thyroid carcinoma: Risk factors and outcome. Surgery.

[147] Rosove M.H., Peddi P.F., Glaspy J.A. (2013). BRAF V600E inhibition in anaplastic thyroid cancer. N. Engl. J. Med..

[148] Logan C.Y., Nusse R. (2004). The Wnt signaling pathway in development and disease. Annu. Rev. Cell Dev. Biol..

[149] Takemaru K.-I., Ohmitsu M., Li F.-Q. (2008). An oncogenic hub: Beta-catenin as a molecular target for cancer therapeutics. Handb. Exp. Pharmacol..

[150] Ha H.T., Lee J.S., Urba S., Koenig R.J., Sisson J., Giordano T., Worden F.P. (2010). A phase II study of imatinib in patients with advanced anaplastic thyroid cancer. Thyroid.

[151] Bible K.C., Suman V.J., Menefee M.E., Smallridge R.C., Molina J.R., Maples W.J., Karlin N.J., Traynor A.M., Kumar P., Goh B.C. (2012). A multiinstitutional phase 2 trial of pazopanib monotherapy in advanced anaplastic thyroid cancer. J. Clin. Endocrinol. Metab..

[152] Savvides P., Nagaiah G., Lavertu P., Fu P., Wright J.J., Chapman R., Wasman J., Dowlati A., Remick S.C. (2013). Phase II trial of sorafenib in patients with advanced anaplastic carcinoma of the thyroid. Thyroid.

[153] Hogan A.R., Zhuge Y., Perez E.A., Koniaris L.G., Lew J.I., Sola J.E. (2009). Pediatric thyroid carcinoma: Incidence and outcomes in 1753 patients. J. Surg. Res..

[154] Donis-Keller H., Dou S., Chi D., Carlson K.M., Toshima K., Lairmore T.C., Howe J.R., Moley J.F., Goodfellow P., Wells S.A.J. (1993). Mutations in the RET proto-oncogene are associated with MEN 2A and FMTC. Hum. Mol. Genet..

[155] Frank-Raue K., Rondot S., Raue F. (2010). Molecular genetics and phenomics of RET mutations: Impact on prognosis of MTC. Mol. Cell. Endocrinol..

[156] Eng C., Clayton D., Schuffenecker I., Lenoir G., Cote G., Gagel R.F., van Amstel H.K., Lips C.J., Nishisho I., Takai S.I. (1996). The relationship between specific RET proto-oncogene mutations and disease phenotype in multiple endocrine neoplasia type 2. International RET mutation consortium analysis. JAMA.

[157] Mulligan L.M., Kwok J.B., Healey C.S., Elsdon M.J., Eng C., Gardner E., Love D.R., Mole S.E., Moore J.K., Papi L. (1993). Germ-line mutations of the RET proto-oncogene in multiple endocrine neoplasia type 2A. Nature.

[158] Romei C., Mariotti S., Fugazzola L., Taccaliti A., Pacini F., Opocher G., Mian C., Castellano M., degli Uberti E., Ceccherini I. (2010). Multiple endocrine neoplasia type 2 syndromes (MEN 2): Results from the ItaMEN network analysis on the prevalence of different genotypes and phenotypes. Eur. J. Endocrinol..

[159] Moline J., Eng C. (2011). Multiple endocrine neoplasia type 2: An overview. Genet. Med..

[160] Moura M.M., Cavaco B.M., Pinto A.E., Domingues R., Santos J.R., Cid M.O., Bugalho M.J., Leite V. (2009). Correlation of RET somatic mutations with clinicopathological features in sporadic medullary thyroid carcinomas. Br. J. Cancer.

[161] Fugazzola L., De Leo S., Perrino M. (2013). The optimal range of RET mutations to be tested: European comments to the guidelines of the American Thyroid Association. Thyroid Res..

[162] Wells S.A., Asa S.L., Dralle H., Elisei R., Evans D.B., Gagel R.F., Lee N., Machens A., Moley J.F., Pacini F. (2015). Revised American Thyroid Association guidelines for the management of medullary thyroid carcinoma. Thyroid.

[163] Wiench M., Wygoda Z., Gubala E., Wloch J., Lisowska K., Krassowski J., Scieglinska D., Fiszer-Kierzkowska A., Lange D., Kula D. (2001). Estimation of risk of inherited medullary thyroid carcinoma in apparent sporadic patients. J. Clin. Oncol..

[164] Oczko-Wojciechowska M., Pfeifer A., Rusinek D., Pawlaczek A., Zebracka-Gala J., Kowalska M., Kowal M., Swierniak M., Krajewska J., Gawlik T. (2015). The prevalence of somatic RAS mutations in medullary thyroid cancer—A Polish population study. Endokrynol. Pol..

[165] Elisei R., Cosci B., Romei C., Bottici V., Renzini G., Molinaro E., Agate L., Vivaldi A., Faviana P., Basolo F. (2008). Prognostic Significance of Somatic *RET* Oncogene Mutations in Sporadic Medullary Thyroid Cancer: A 10-Year Follow-Up Study. J. Clin. Endocrinol. Metab..

[166] Sherman S., Elisei R., Mueller S., Schoffski P., Brose M., Shah M., Licitra L., Jarzab B., Medvedev V., Kreissl M. The Impact of RET and RAS Mutation Status on Overall Survival in the EXAM Trial, a Phase 3 Study of Cabozantinib in Patients with Progressive, Metastatic Medullary Thyroid Cancer (MTC). Proceedings of the ITC Annual Meeting.

[167] Schlumberger M., Pacini F., Tuttle R. (2016). Medullary thyroid carcinoma. Thyroid Tumors.

[168] Kloos R.T., Eng C., Evans D.B., Francis G.L., Gagel R.F., Gharib H., Moley J.F., Pacini F., Ringel M.D., Schlumberger M. (2009). Medullary thyroid cancer: Management guidelines of the American Thyroid Association. Thyroid.

[169] Jarząb B., Król A., Hasse-Lazar K., Jurecka-Lubieniecka B. (2013). Presentation of points of general discussion and voting among the speakers of the European Thyroid Association-Cancer Research Network (ETA-CRN) meeting in Lisbon, 2009, entitled “European comments to ATA medullary thyroid cancer guidelines”. Thyroid Res..

[170] Jarzab B., Szpak-Ulczok S., Wloch J., Czarniecka A., Krajewska J. (2013). Timing and criteria for prophylactic thyroidectomy in asymptomatic RET carriers—The role of Ct serum level. Thyroid Res..

[171] Elisei R., Pinchera A. (2012). Advances in the follow-up of differentiated or medullary thyroid cancer. Nat. Rev. Endocrinol..

[172] Modigliani E., Cohen R., Campos J.M., Conte-Devolx B., Maes B., Boneu A., Schlumberger M., Bigorgne J.C., Dumontier P., Leclerc L. (1998). Prognostic factors for survival and for biochemical cure in medullary thyroid carcinoma: Results in 899 patients. The GETC Study Group. Groupe d’étude des tumeurs à calcitonine. Clin. Endocrinol. (Oxf.).

[173] Wygoda Z., Oczko-Wojciechowska M., Gubała E., Pawlaczek A., Kula D., Wiench M., Włoch J. (2006). Medullary thyroid carcinoma: The comparison of the hereditary and sporadic types of cancer. Endokrynol. Pol..

[174] Gawlik T., d’Amico A., Szpak-Ulczok S., Skoczylas A., Gubala E., Chorazy A., Gorczewski K., Wloch J., Jarzab B. (2010). The prognostic value of tumor markers doubling time in medullary thyroid carcinoma-preliminary report. Thyroid Res..

[175] Meijer J.A.A., le Cessie S., van den Hout W.B., Kievit J., Schoones J.W., Romijn J.A., Smit J.W.A. (2010). Calcitonin and carcinoembryonic antigen doubling times as prognostic factors in medullary thyroid carcinoma: A structured meta-analysis. Clin. Endocrinol..

[176] Kouvaraki M.A., Shapiro S.E., Fornage B.D., Edeiken-Monro B.S., Sherman S.I., Vassilopoulou-Sellin R., Lee J.E., Evans D.B. (2003). Role of preoperative ultrasonography in the surgical management of patients with thyroid cancer. Surgery.

[177] Pellegriti G., Leboulleux S., Baudin E., Bellon N., Scollo C., Travagli J.P., Schlumberger M. (2003). Long-term outcome of medullary thyroid carcinoma in patients with normal postoperative medical imaging. Br. J. Cancer.

[178] Wells S.A., Robinson B.G., Gagel R.F., Dralle H., Fagin J.A., Santoro M., Baudin E., Elisei R., Jarzab B., Vasselli J.R. (2012). Vandetanib in patients with locally advanced or metastatic medullary thyroid cancer: A randomized, double-blind phase III trial. J. Clin. Oncol..

[179] Elisei R., Schlumberger M.J., Müller S.P., Schöffski P., Brose M.S., Shah M.H., Licitra L., Jarzab B., Medvedev V., Kreissl M.C. (2013). Cabozantinib in progressive medullary thyroid cancer. J. Clin. Oncol..

[180] Schlumberger M., Bastholt L., Dralle H., Jarzab B., Pacini F., Smit J.W.A. (2012). European thyroid association guidelines for metastatic medullary thyroid cancer. Eur. Thyroid J..

